# *De novo* copy number variations in candidate genomic regions in patients of severe autism spectrum disorder in Vietnam

**DOI:** 10.1371/journal.pone.0290936

**Published:** 2024-03-07

**Authors:** Hoa Thi Phuong Bui, Duong Huy Do, Ha Thi Thanh Ly, Kien Trung Tran, Huong Thi Thanh Le, Kien Trung Nguyen, Linh Thi Dieu Pham, Hau Duc Le, Vinh Sy Le, Arijit Mukhopadhyay, Liem Thanh Nguyen

**Affiliations:** 1 High Technology Center, Vinmec Health Care System, Hai Ba Trung, Ha Noi, Vietnam; 2 Translational Medicine Laboratory, Biomedical Research Centre, University of Salford, Salford, United Kingdom; 3 Vinmec Research Institute of Stem Cell and Gene Technology, Vinmec Health Care System, Hai Ba Trung, Ha Noi, Vietnam; 4 Big Data Institute, Vinmec Health Care System, Hai Ba Trung, Ha Noi, Vietnam; 5 University of Engineering and Technology, Vietnam National University Hanoi, Cau Giay, Hanoi, Vietnam; Shaheed Rajaei Hospital: Rajaie Cardiovascular Medical and Research Center, ISLAMIC REPUBLIC OF IRAN

## Abstract

Autism spectrum disorder (ASD) is a developmental disorder with a prevalence of around 1% children worldwide and characterized by patient behaviour (communication, social interaction, and personal development). Data on the efficacy of diagnostic tests using copy number variations (CNVs) in candidate genes in ASD is currently around 10% but it is overrepresented by patients of Caucasian background. We report here that the diagnostic success of *de novo* candidate CNVs in Vietnamese ASD patients is around 6%. We recruited one hundred trios (both parents and a child) where the child was clinically diagnosed with ASD while the parents were not affected. We performed genetic screening to exclude RETT syndrome and Fragile X syndrome and performed genome-wide DNA microarray (aCGH) on all probands and their parents to analyse for *de novo* CNVs. We detected 1708 non-redundant CNVs in 100 patients and 118 (7%) of them were *de novo*. Using the filter for known CNVs from the Simons Foundation Autism Research Initiative (SFARI) database, we identified six CNVs (one gain and five loss CNVs) in six patients (3 males and 3 females). Notably, 3 of our patients had a deletion involving the *SHANK3* gene–which is the highest compared to previous reports. This is the first report of candidate CNVs in ASD patients from Vietnam and provides the framework for building a CNV based test as the first tier screening for clinical management.

## Introduction

Autism spectrum disorder (ASD) is one of the most prevalent disorders in children. It was estimated that about 1.7% (1 in 59 children) in the United States [1] are diagnosed with ASD. As a complex disorder, the phenotype and severity of ASD can vary. The disorder typically affects social interaction and adaptability, which can have serious implications for the development of a child. Different studies have shown evidences that ASD is a result of complex interactions between genetics and the environment, with a strong genomic contribution [2, 3]. Microarray-based comparative genomic hybridization (aCGH) is one of the effective genomic methods for analysis of copy number variations and structural alterations. Genomic microarrays for CNV analysis is now recommended as the first tier test for patients with neurocognitive disorders including ASD, intellectual disability, and development delays [4]. The diagnostic yield of this technology depends not only on the population studied but also on the specific aCGH platforms used. Based on case-control study design, overall diagnostic yield of aCGH is around 10%, ranging between 8.7% - 15.6% [5–8]. The majority of these studies are focused on patients of Caucasian ethnicity, while a few of them are from Asian countries such as China, Korea, Japan, Hongkong and Thailand [9–12]. To date, there is no published studies on genetics of ASD in the Vietnamese population–we aim to bridge that gap through this study. Identification of population specific genetic alterations is crucial for diagnosis, prognosis, genetic counseling and potential prenatal diagnosis for families with autistic children. While ASD could have genetic contributions by a combination of *de novo* and inherited variations, *de novo* variations are likely more important as most patients are sporadic [13, 14]. *De novo* CNVs may in fact, explain some of the “missing heritability” of ASD [15], and so we focused on trios for reliable detection of *de novo* CNVs.

The pathophysiology and mechanisms of autism have not been thoroughly clarified. Among many theories, neural hypoperfusion and immune dysregulations have been reported to be the two major physiological alterations and are correlated with the severity of autism symptoms [16]. Whole bone marrow mononuclear cells (BMMNCs) which consist of both hematopoietic stem cells (HSCs) and Mesenchymal stem cells (MSCs) could potentially produce a better and synergistic effect as compared to each cell line alone [17, 18]. Some studies have shown promising results suggesting that stem cell therapy could be an additional treatment for autism [19–21], however further research is needed to demonstrate the safety and effectiveness of this approach.

## Methods

### Patient recruitment–Sample collection

One hundred Vietnamese Children in Vinmec International Hospital, Vietnam, were diagnosed with autism spectrum disorder by Diagnostic and Statistical Manual of Mental Disorders (DSM) version 4 or 5, Autism Diagnostic Observation Schedule (ADOS) and Childhood Autism Rating Scale (CARS). All patients were diagnosed based on three criteria above by physicians trained for ASD diagnosis at Vinmec International Hospital during the period 1^st^ January 2017 until 31^st^ December 2018. Inclusion criteria for cases were: (i) met autism spectrum disorder diagnoses by above three tools, and (ii) CARS score 35 points or above. This research was carried out in accordance with the World Medical Association’s ‘Declaration of Helsinki’. This study was approved by the ethical review board for biomedical research in Vinmec International General Hospital JSC, Vietnam and Ethics committee of University of Salford, Manchester, United Kingdom. Following a description of the study, written informed consent was obtained from the guardian of each patient as well as from their parents. After the medical examination, the individual identifiers (name, address etc) were removed from the samples and the records were anonymized for all authors involved.

### DNA isolation and qualification

Peripheral blood (3–4 ml in EDTA) was collected from the patients and their unaffected parents for DNA extraction. QIAamp Blood Kit (Qiagen, Hilden, Germany) was used for genomic DNA extraction. The concentration of DNA in all samples was measured by Nanodrop spectrophotometer. All samples had an A_260_/A_280_ ratio of at least 1.8 and minimum concentration of DNA was 20 ng/ul. An example of gel electrophoresis checking for DNA quality is given in the supporting information (S1 Fig in S1 File).

### Fragile X and MeCP2 DNA test

All patients were excluded for Fragile X syndrome by examining trinucleotide repeat expansion and the methylation status of *FMR1* gene. All female patients were tested (Sanger sequencing) for mutations in exons 2, 3 and 4 of the *MECP2* gene.

### Microarray-based comparative genomic hybridization

We performed aCGH experiments on SurePrint G3 Human CGH Microarray, 2x400K KIT (Agilent) to detect CNVs. The experimental and analysis protocol were as advised by the manufacturer. The purified, labelled DNA was checked on a Nanodrop with specific Activity of Cyanine 3 and Cyanine 5 Labeled Sample (pmol/ μg) from 20 to 60. Each specimen sample was mixed together with the corresponding commercial reference sample provided in the Kit. Then, samples were hybridized with Agilent slides at 67°C for 40 hours. After washing, the slide was scanned by Agilent Microarray Scanner. Agilent Feature Extraction Software 11.5.1.1 was used to assess the quality of image data file. The first-pass quality control (QC) filter of <0.23 derivative log-ratio threshold was applied.

The data-quality was checked in 4 steps, (i) input DNA quality in agarose gel (ii) digestion of DNA after restriction digestion by agarose gel, (iii) labeling efficiency by Nanodrop, and (iv) the quality of aCGH data after analysis by the Cytogenomics software provided by Agilent.

### Copy number variations calling and annotation

We used ADM-2 algorithm to call CNVs with the following criteria: (1) up to 6.0 standard deviations, (2) three consecutive probes, (3) the minimum absolute log ratio of 0.25 (the default in ADM-2), (4) FuzzyZerri = ON, and (5) Diploid Peak Centralization: ON. We also removed regions of called CNVs that belong to gain/loss regions in the Agilent.

A list of candidate genes for further analysis were obtained using the following criteria: (1) containing at least one exon overlapped with called CNVs, (2) not belonging to the Database of Genomic Variants stringent map as CNVs that are considered as benign (http://dgv.tcag.ca/dgv/app/downloads); and (3) not belonging to any CNVs with frequency ≥1% in the NSTD100 database (https://www.ncbi.nlm.nih.gov/dbvar/studies/nstd100/). It is possible that CNVs with frequency >1% may contribute as weak risk factors but such events are typically smaller and have not been sufficiently detected by microarrays [22].

Candidate genes were then annotated with the following information: (1) The number of exons that overlapped with called CNVs, (2) Phenotype and inheritance model obtained from the Clinical Genome Database, (3) DGV stringent map [23], (4) DGV standard CNV database [24], (5) Pathogenic regions in the ISCA database (NSTD45), (6) 55 pathogenic regions in the NSTD1000, and (7) Information obtained from SFARI and DECIPHER databases.

### Copy number variation interpretation

A CNV was annotated as pathogenic if it covers known pathogenic regions in either ISCA or NSTD100 databases.

*De novo* CNVs are defined as observed in the patients but not present in their parents. If the *de novo* CNV included a candidate gene annotated as highly likely pathogenic, it was considered as highly likely *de novo* pathogenic CNV.

### Copy number variation validation

Quantitative real-time PCR (qPCR) was performed to validate CNVs. *VPS29* gene was selected as the reference gene (endogenous control) for normalization purposes due to the absence of any reported CNV in this gene. Primer were designed, and qPCR using SYBR green PCR Kit (Qiagen, Germany) was performed on a 7500 Real-time PCR System (Applied Biosystems, MA). Quantification of target sequence was normalized, and relative copy number (RCN) determined based on comparative ΔΔCt method with a normal control DNA as the calibrator. The ΔΔCt was calculated as follows: ΔΔCt = (ΔCt unknown sample-ΔCt control sample). Normalized copy number = 2^ˉΔΔCt^. A 0.5-fold RCN was selected as a loss (heterozygous deletion), and above 1.5-fold RCN was chosen as a gain [from 3 copies] [25]. The details of all primer sequences are available in the supporting information (S5 Table in S1 File).

## Results

### Clinical characterization of patients

One hundred autistic children and their unaffected parents were recruited for the study including two families with 2 affected siblings (dizygotic twins). The patients were unrelated, as determined by their reported family structures. Four patients had an uncertainty about their paternal origin (ASD080, ASD091, ASD100 and ASD101 [26]. All patients were confirmed negative with Fragile X test and all females were negative for *MECP2* mutation.

Out of the 100 Vietnamese autism patients in our study, 83 were male and 17 were female, with the male to female ratio 5:1. Their age ranged between 3 to 18 years old (average age was 6.91 years). The average age at diagnosis is 25.57 months, with 77 patients diagnosed as an autism suspect before 36 months of age. The DSM result showed that 79/97 patients were classified as level 3 –“requiring very substantial support” and the CARS tool helped group 95/98 patients with severe autism with scores between 35 and 55.5 (average score of 46.8). Patient and family histories were used to identify any other neurodevelopmental or genetic disorders. In 100 probands, 14 participants have secondary diagnosis including Intellectual Disability, epilepsy and Cerebral Palsy. Four families had a positive history of ASD; and 23 others had related neuro-mental disorders (all details in Table 1 and the clinical features in the supporting information (S1 and S2 Tables in S1 File).

**Table 1:** Clinical features of 100 probands.

Domain	Male	Female
Median age (years)	10.5 (range: 3–18 years old)	
Gender	83	17
***CARS (98/100)***		
Severe	79	16
Mild-Moderate	2	1
***DSM (97/100)***		
Level 3	69	10
Level 1–2	13	5
***Secondary diagnosis***		
Intellectual disability (ID)	7	2
Epilepsy	2	2
Cerebral palsy	1	0
None	73	13
***Family history (up to 3***^***rd***^ ***cousin*, *excludes parents)***		
Normal	64	13
Autism	4	0
Other neurodevelopmental disorders (for example ID, Epilepsy, Cerebral palsy, Parkinson and etc)	15	4

**Fig 1:**
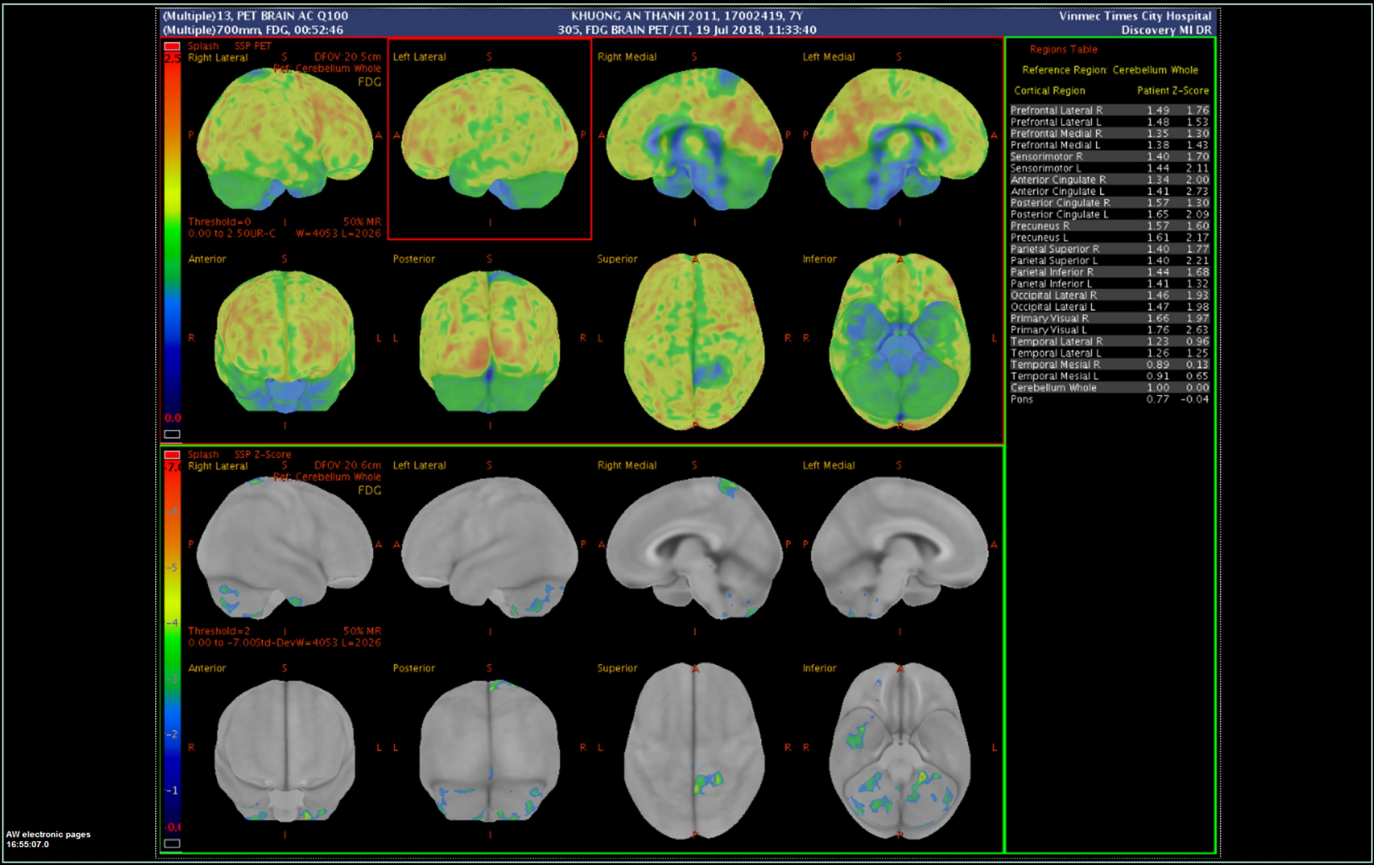
A representative example of imaging of Autism spectrum disorder (ASD) patient’s brain. The Positron emission tomography-computed tomography (PET-CT) images show hypometabolism in multiple areas of the brain of the ASD patient. Dark blue brain areas were defined as severe reductions in FDG metabolic rate and light blue brain areas were assessed as moderate metabolism. Green was considered to have mild metabolic reduction. The yellow-orange brain region was assessed as increased FDG metabolism.

Twenty-nine out of 100 patients underwent dedicated 18F-fluorodeoxyglucose (18F-FDG) brain positron emission tomography-computed tomography (PET-CT). On post hoc analysis, the altered FDG metabolism regions in autistic children were evaluated based on a normal distribution curve. A value lower than the standard deviation (SD) from the mean value of the SUV was considered to be hypometabolic; whereas a value greater than SD was considered hypermetabolic [27]. Evaluation of PET-CT images: Dark blue brain areas were defined as severe reductions in FDG metabolic rate and light blue brain areas were assessed as moderate metabolism. Green was considered to have mild metabolic reduction. The yellow-orange brain region was assessed as increased FDG metabolism [28]. All of the PET-CT images in 29 patients showed hypometabolism in the brain with nine patients marked as a severe level of hypometabolism, 16 patients demonstrated moderate metabolism and four patients showed a mild level. Each patient showed variations in one or more parts of their brain, one or both sides with most of them showing hypometabolism in temporal lobe, parietal lobe, frontal lobe, hippocampus and limbic cortex in both sides of the brain. An example of PET-CT image is shown in Fig 1 and detail of MRI and PET-CT of patients is shown in the supporting information (S3 Table in S1 File).

### Summary of aCGH experiment and QPCR validation

All 300 samples passed the QC as high quality using the quality control metrics of cytogenetics software.

#### The distribution of inherited and *de novo* CNVs

**Fig 2:**
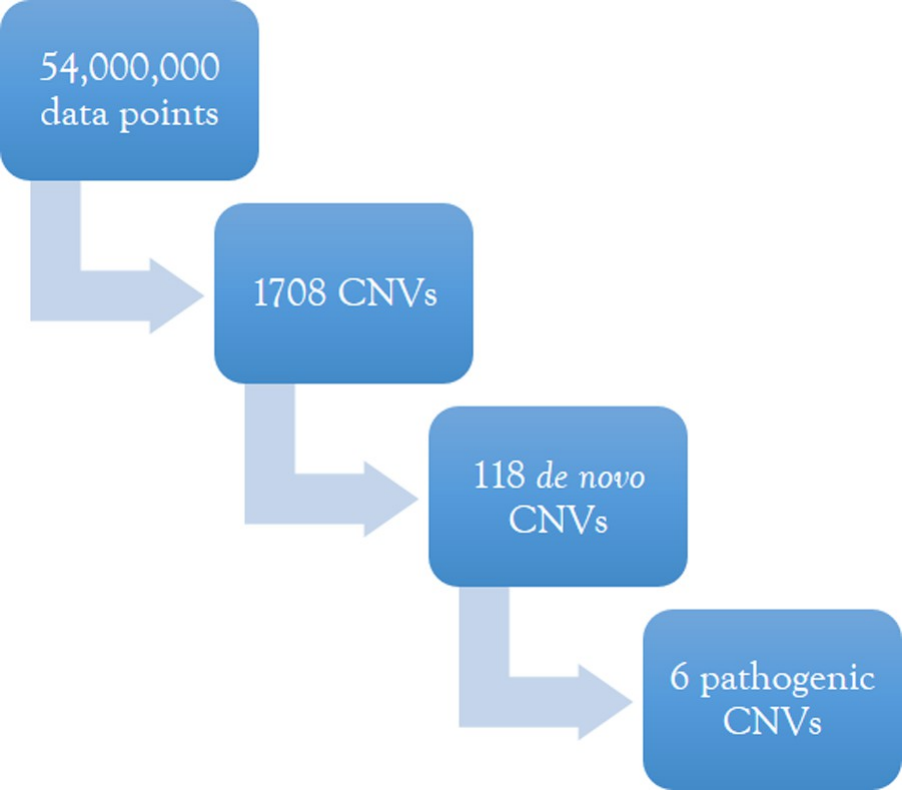
Data summary of *de novo* copy number variations (CNVs) in 100 ASD patients. A total of 54 million data points were analysed. 1708 on-redundant CNVs were found in 100 patients of which 118 were *de novo* after removing inherited CNVs from at least one parent. Based on the biggest autism database (SFARI) and the guideline from American College of Medical Genetics and Genomics (ACMG), 6 CNVs out of 118 were classified as pathogenic related to autism.

We obtained 1708 distinct/non-redundant CNVs of which 118 CNVs were *de novo*. Out of the 118 non-redundant *de novo* CNVs, six were pathogenic, 13 were variants of uncertain significance (VUS) and 99 were benign (Fig 2 and details of 118 non-redundant *de novo* CNVs are shown in the supporting information (S4 Table in S1 File). In six patients (three females and three males), we identified six pathogenic *de novo* CNVs which were one duplication and five deletions. All identified *de novo* CNVs were classified according to the ACMG Guidelines [29]. A summary table of all pathogenic CNVs is shown in Table 2.

**Table 2:** Details of candidate *de novo* CNVs. All candidate CNVs were found by aCGH testing on 100 trios and are mentioned in the SFARI database. Included are patient ID, position of CNV in chromosome, the size and aberration and also related genes (GRCh37/hg19).

Patients ID and gender	Chromosome region	Start point	End point	Size (bp)	Aberration type	Representative gene within the CNV
ASD025 (F)	11q13.2-q13.4	67,856,136	73,062,386	5,206,250	Loss	*SHANK2*
ASD047 (M)	22q13.32-q13.33	48,746,241	51,178,264	2,432,023	Loss	*SHANK3*
ASD080 (F)	22q13.33	51,137,326	51,170,223	32,897	Loss	*SHANK3*
ASD089 (M)	22q13.33	49,564,639	51,178,264	1,613,625	Loss	*SHANK3*
ASD091 (F)	4q34.1-q35.2	175,897,368	190,896,674	14,999,306	Loss	*LRP2BP*
ASD097 (M)	15q11.1-q13.3	20,102,541	32,445,252	12,342,711	Gain	*GABRB3*

#### 22q13.32-q13.33 deletion (candidate gene *SHANK3*)

Three patients (ASD047, ASD080 and ASD089) had deletion CNVs containing the *SHANK3* gene and defined as pathogenic for ASD (Scored 1s in SFARI gene scoring). Details of CNV position are described in the supporting information (S2 Fig in S1 File). These CNVs were absent in all 200 unaffected parents–suggesting its pathogenic role. Specifically, patient ASD047 is an 8-year-old boy born from a normal pregnancy and delivery history, diagnosed with autism (ICD-10: F84.0 –DSM 5 level 3, ADOS 22/12 and CARS 49.5) and Intellectual Disability (ID) (ICD-10: F70). He went to the Pediatric National Hospital at 8 months with ID signs. He currently has poor communication skills defined by poor eye contact, expressionless face, lack of expressive speech and limit in daily self-care. He was found to have a *de novo* 2.43 Mb deletion CNV which contains *SHANK3* gene.

Patient ASD080 is a 5-year-old girl diagnosed with autism (ICD-10: F84.0 –DSM 5 level 3, ADOS 21/12 and CARS 49.5) with normal family history. Her mother had a normal pregnancy, and she was born at 40 weeks of gestation without any complications. The proband showed significant psychomotor development delay from birth but she was able to walk at 2 years of age. Currently, she can only use a few single words, has limited social interaction and tends to play alone, shows no response to her name and lacks eye contact. She has hyperactivity and numerous repetitive actions. PET-CT image demonstrates hypometabolism in the frontal lobe, parietal lobe, hippocampus and limbic cortex. The array CGH test has found a 32 Kb deletion which encompasses 9/25 exons of *SHANK3* gene in this patient. This patient was reported as having an unclear biological relationship with her father and this CNV was not found in her biological mother.

The patient ASD089 is a 5-year-old boy diagnosed with autism (ICD-10: F84.0 –DSM 5 level 3, ADOS 20/12 and CARS 54) with normal maternal pregnancy and family history. He had asphyxiation after birth (no Apgar score information). He could walk unsupported at 2 years of age. He showed lack of expressive speech, limited social communication and cannot do basic self-care. He also showed hyperactivity with restricted interests such as turning lights on-off light and opening-closing doors. He was found to have a 1.61 Mb deletion containing the entire *SHANK3* gene.

#### 4q34.1-q35.2 deletion (candidate gene *LRP2BP*)

This *de novo* CNV was only present in patient ASD091 and absent in all 200 unaffected parents.

Patient ASD091 is an 8-year-old girl diagnosed with autism (ICD-10: F84.0 –DSM 5 level 3, ADOS 22/12 and CARS 51) from a normal family history. Her mother had to take hormonal drugs for a risk of miscarriage at 8 weeks of pregnancy and caesarean delivery due to early amniotomy at 36 weeks of gestation. She currently shows lack of verbal communication, eye contact and other social interactions. She also has hyperactivity, self-harms and restricted activity such as striking toys to her teeth or to the floor and biting. She is unable to do basic self-care. The patient was found to have a 14 Mb deletion CNV at 4q34.1-q35.2 which overlaps with *LRP2BP*–a candidate gene for ASD. This individual has the same concern about biological relationship with her father as the patient ASD080. But the mother does not have this CNV.

#### 11q13.2-q13.4 deletion (candidate genes *SHANK2*)

This *de novo* CNV was only detected in patient ASD025.1, a 6-year-old girl diagnosed with autism (ICD-10: F84.0 –DSM 5 level 3, ADOS 17/12 and CARS 45.5) and ID (ICD-10: F70) with a normal family history. Her mother suffered from Rubella infection one month before pregnancy. The child was born with a birth weight of 2800 gm without any complications at 38 weeks. She had flat feet and showed psychomotor delay, only walking at 2 years, babbling at 3 years of age and reduced gross and fine motor skills. Currently she lacks speech as well as non-verbal communication and also demonstrates repetitive activity like turning around a wheel and propellers. A 5 Mb *de novo* deletion CNV containing many genes, amongst them, *SHANK2* gene being the most significant candidate for ASD, was identified.

#### A gain at 15q11.1-q13.3 (candidate gene *GABRB3*)

Patient ASD097 had this *de novo* CNV which was not present in any other samples in this study (including all unaffected parents).

Patient ASD097 is a 13-year-old boy diagnosed with autism (ICD-10: F84.0 –DSM 5 level 3, ADOS 22/12 and CARS 52) and ID (ICD-10: F70). The mother had a normal pregnancy, delivery and family history. Psychomotor development was significantly delayed from birth, but he was able to walk unsupported at 2 years of age. Currently, he presents with social interaction disorders, lacks expressive speech and produces inarticulate sounds. He exhibits numerous repetitive behaviours and hyperactivity. The patient has a 12 Mb *de novo* gain containing many genes reported in the ASD database and covering 2 pathogenic regions (15q13.3 duplication and Prader-Willi syndrome (PWS)/Angelman syndrome (AS)).

**Fig 3:**
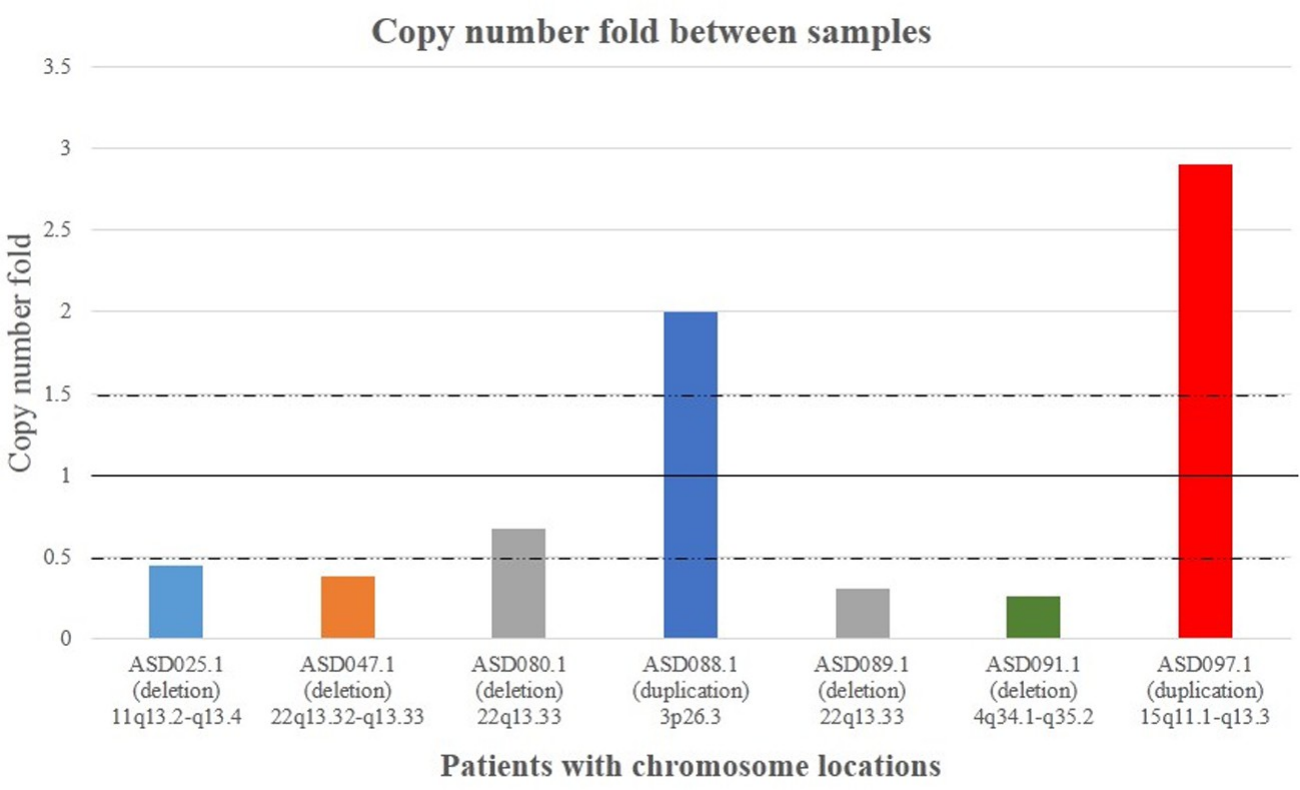
Representative Real-time PCR for array comparative genomic hybridization (aCGH) data validation. The Real-time PCR experiment was applied to validate the aCGH results. Copy number fold shows the number of copies in each ASD patient. A value of 1 in vertical axis indicate the normal scenario with 2 copies. The graph represents the validation of 4 deletion CNVs in 5 different patients and 2 duplication CNVs in 2 participants.

All candidate CNVs are confirmed by Real-time PCR and the results are shown in Fig 3.

## Discussion

This is the first ASD genomics study investigating CNVs in the Vietnamese population. In this project, we focused on the utility of using aCGH to detect *de novo* CNV for the potential genetic diagnostic of autism. We found that in our cohort, 6% of ASD patients can be diagnosed based on *de novo* CNVs in candidate genomic regions. We note that given our study design, any germline CNVs in the parents of the patients will be documented as *de novo* CNV in the patient.

We found 3 ‘unrelated’ patients (ASD047, ASD080 and ASD089) having a heterozygous deletion involving the *SHANK3* gene (S2 Fig in S1 File); putting the *de novo* deletion frequency of *SHANK3* at 3%. *SHANK3* encodes a scaffold protein in the postsynaptic densities of excitatory synapses, which plays a role in the connection of membrane-bound receptors to the actin cytoskeleton. Loss of one functional copy (haploinsufficiency) of *SHANK3* gene is known to have a causative role in a monogenic form of ASD with a frequency of 0.5% [30]. Although *SHANK3* CNVs are well known contributors in ASD, to our knowledge 3% is the highest frequency reported so far. In European, American, and Australian populations the *SHANK3* CNV frequency is reported to be much lower (0.24%) while in the Chinese population it is around 1.7% [7, 31].

Shank/ProSAP proteins encoded by the three genes *SHANK1*, *SHANK2* and *SHANK3* are master scaffold proteins, located at the post-synaptic density of glutamatergic synapses, essential for synaptic development and functions. There is strong evidence supporting the association between this family of genes and ASD, although each gene could affect the patient differently [32]. The Phelan-McDermid syndrome (PMS) was the first pathology report in patients with an alteration in *SHANK* genes [33]. This syndrome is described as a neurological deficit characterized by global developmental delay, moderate to severe intellectual impairment, absent or severely delayed speech, and neonatal hypotonia. And, over 50% of patients with this disorder have autism or autistic-like behavior [7]. Phelan-McDermid syndrome is caused by deletion or changing in chromosomal structure in 22q13 region or mutation in *SHANK3* gene [34]. In our study, the 3 patients with *SHANK3* gene deletion could be also diagnosed as patients of Phelan-McDermid syndrome. Broadly, an increased frequency of *SHANK3* CNVs in ASD patients in our study and in Chinese population suggest a higher frequency of Phelan-McDermid syndrome in DD/MR/ASD patients among East Asians [34].

In recent research, mutations in *SHANK1* and *SHANK2* have also been involved with ASD [35], supporting the idea that there is a general function for these proteins working within common molecular pathways associated with ASD [36]. In our study, we also report a case with deletion in 11q13.2–13.4 region containing *SHANK2* gene. Different independent studies also supported evidence for the involvement of *SHANK2* in ASD and ID [37–42]. It was reported that *SHANK2* variants can reduce the number of synapses *in vitro*.

Increasing awareness and knowledge about ASD and commonly related neurobehavioral conditions with the contribution of genetic differences will be especially useful for healthcare professionals who provide evaluation and treatment services for ASD. This will also be meaningful for individuals living with ASD, their families and possibly for the benefit of society overall. Early recognition, diagnosis, and treatment could help individuals with ASD achieve optimal long-term outcomes and improved quality of life. Understanding of genetic patterns which could affect treatment outcomes, for example medications or stem cell transplantation, will help to improve the overall health of patients. Our group has recently reported encouraging initial results from stem cell transplantations in ASD patients [43]. Future studies aiming at specific therapeutic regimes based on their genetic findings will pave novel therapeutic routes for ASD management in Vietnam.

## Conclusion

Our study shows that Vietnamese ASD patients from a *de novo* CNV perspective have similar genomic features and so CNV analysis can potentially be used as a first-tier screening test for clinical assessment. Also, with a limited number of samples, we found the highest (3%) contribution of deletions involving *SHANK3* gene. Given the current limitations of the healthcare system and general economic status of people in Vietnam, genome wide screens are realistically not possible as a standard screening for ASD diagnosis. With such limitations, a simple and cost-effective PCR based test for SHANK3 deletion can be an effective tool for screening–especially for patients showing Phelan-McDermid syndrome (depending on examination results from neuropsychiatrist).

## Supporting information

S1 File (DOCX)

## References

[1] CDC. Why Act Early if You’re Concerned about Development? 2020 [cited 2020 9th september]. Available from: https://www.cdc.gov/ncbddd/actearly/whyActEarly.html.

[2] Chaste P, Leboyer M. Autism risk factors: genes, environment, and gene-environment interactions. Dialogues Clin Neurosci. 2012;14(3):281–92. doi: 10.31887/DCNS.2012.14.3/pchaste 23226953 PMC3513682

[3] Yoo H. Genetics of Autism Spectrum Disorder: Current Status and Possible Clinical Applications. Exp Neurobiol. 2015;24(4):257–72. doi: 10.5607/en.2015.24.4.257 26713075 PMC4688327

[4] Kearney HM, Thorland EC, Brown KK, Quintero-Rivera F, South ST. American College of Medical Genetics standards and guidelines for interpretation and reporting of postnatal constitutional copy number variants. Genetics in medicine: official journal of the American College of Medical Genetics. 2011;13(7):680–5. doi: 10.1097/GIM.0b013e3182217a3a 21681106

[5] Moreira ES, Silva IMW, Lourenço N, Moreira DP, Ribeiro CM, Martins ALB, et al. Detection of small copy number variations (CNVs) in autism spectrum disorder (ASD) by custom array comparative genomic hybridization (aCGH). Research in Autism Spectrum Disorders. 2016;23:145–51.

[6] Siu WK, Lam CW, Mak CM, Lau ET, Tang MH, Tang WF, et al. Diagnostic yield of array CGH in patients with autism spectrum disorder in Hong Kong. Clinical and translational medicine. 2016;5(1):18. doi: 10.1186/s40169-016-0098-1 27271878 PMC4896892

[7] Shin S, Yu N, Choi JR, Jeong S, Lee K-A. Routine Chromosomal Microarray Analysis is Necessary in Korean Patients With Unexplained Developmental Delay/Mental Retardation/Autism Spectrum Disorder. Annals of Laboratory Medicine. 2015;35(5):510–8. doi: 10.3343/alm.2015.35.5.510 26206688 PMC4510504

[8] Stobbe G, Liu Y, Wu R, Hudgings LH, Thompson O, Hisama FM. Diagnostic yield of array comparative genomic hybridization in adults with autism spectrum disorders. Genetics in medicine: official journal of the American College of Medical Genetics. 2014;16(1):70–7. doi: 10.1038/gim.2013.78 23765050

[9] Liu X, Shimada T, Otowa T, Wu YY, Kawamura Y, Tochigi M, et al. Genome-wide Association Study of Autism Spectrum Disorder in the East Asian Populations. Autism Res. 2016;9(3):340–9. doi: 10.1002/aur.1536 26314684

[10] Mak ASL, Chiu ATG, Leung GKC, Mak CCY, Chu YWY, Mok GTK, et al. Use of clinical chromosomal microarray in Chinese patients with autism spectrum disorder—implications of a copy number variation involving DPP10. Molecular Autism. 2017;8(1):31. doi: 10.1186/s13229-017-0136-x 28670437 PMC5485587

[11] Cho S-C, Yoo HJ, Park M, Cho IH, Kim B-N, Kim J-W, et al. Genome-wide association scan of korean autism spectrum disorders with language delay: a preliminary study. Psychiatry investigation. 2011;8(1):61–6. doi: 10.4306/pi.2011.8.1.61 21519539 PMC3079188

[12] Hnoonual A, Thammachote W, Tim-Aroon T, Rojnueangnit K, Hansakunachai T, Sombuntham T, et al. Chromosomal microarray analysis in a cohort of underrepresented population identifies SERINC2 as a novel candidate gene for autism spectrum disorder. Scientific reports. 2017;7(1):12096-. doi: 10.1038/s41598-017-12317-3 28935972 PMC5608768

[13] State MW, Levitt P. The conundrums of understanding genetic risks for autism spectrum disorders. Nature neuroscience. 2011;14(12):1499–506. doi: 10.1038/nn.2924 22037497 PMC3940335

[14] Devlin B, Scherer SW. Genetic architecture in autism spectrum disorder. Current opinion in genetics & development. 2012;22(3):229–37. doi: 10.1016/j.gde.2012.03.002 22463983

[15] Matsunami N, Hadley D, Hensel CH, Christensen GB, Kim C, Frackelton E, et al. Identification of Rare Recurrent Copy Number Variants in High-Risk Autism Families and Their Prevalence in a Large ASD Population. PLOS ONE. 2013;8(1):e52239. doi: 10.1371/journal.pone.0052239 23341896 PMC3544904

[16] Ichim TE, Solano F, Glenn E, Morales F, Smith L, Zabrecky G, et al. Stem cell therapy for autism. Journal of translational medicine. 2007;5:30. doi: 10.1186/1479-5876-5-30 17597540 PMC1914111

[17] Pösel C, Möller K, Fröhlich W, Schulz I, Boltze J, Wagner DC. Density gradient centrifugation compromises bone marrow mononuclear cell yield. PloS one. 2012;7(12):e50293. doi: 10.1371/journal.pone.0050293 23236366 PMC3516517

[18] Brenes RA, Bear M, Jadlowiec C, Goodwin M, Hashim P, Protack CD, et al. Cell-based interventions for therapeutic angiogenesis: review of potential cell sources. Vascular. 2012;20(6):360–8. doi: 10.1258/vasc.2011.201205 23086985 PMC3670829

[19] Lv YT, Zhang Y, Liu M, Qiuwaxi JN, Ashwood P, Cho SC, et al. Transplantation of human cord blood mononuclear cells and umbilical cord-derived mesenchymal stem cells in autism. Journal of translational medicine. 2013;11:196. doi: 10.1186/1479-5876-11-196 23978163 PMC3765833

[20] Sharma A, Badhe P, Gokulchandran N, Kulkarni P, Mishra P, Shetty A, et al. An Improved Case of Autism as Revealed by PET CT Scan in Patient Transplanted with Autologous Bone Marrow Derived Mononuclear Cells. Journal of Stem Cell Research and Therapy. 2013;3.

[21] Sharma A, Gokulchandran N, Sane H, Nagrajan A, Paranjape A, Kulkarni P, et al. Autologous Bone Marrow Mononuclear Cell Therapy for Autism: An Open Label Proof of Concept Study. Stem cells international. 2013;2013:623875. doi: 10.1155/2013/623875 24062774 PMC3767048

[22] Coe BP, Witherspoon K, Rosenfeld JA, van Bon BWM, Vulto-van Silfhout AT, Bosco P, et al. Refining analyses of copy number variation identifies specific genes associated with developmental delay. Nature genetics. 2014;46(10):1063–71. doi: 10.1038/ng.3092 25217958 PMC4177294

[23] Zarrei M, MacDonald JR, Merico D, Scherer SW. A copy number variation map of the human genome. Nature Reviews Genetics. 2015;16(3):172–83. doi: 10.1038/nrg3871 25645873

[24] MacDonald JR, Ziman R, Yuen RKC, Feuk L, Scherer SW. The Database of Genomic Variants: a curated collection of structural variation in the human genome. Nucleic Acids Res. 2014;42(D1):D986–D92. doi: 10.1093/nar/gkt958 24174537 PMC3965079

[25] Hussein IR, Magbooli A, Huwait E, Chaudhary A, Bader R, Gari M, et al. Genome wide array-CGH and qPCR analysis for the identification of genome defects in Williams’ syndrome patients in Saudi Arabia. Mol Cytogenet. 2016;9:65-. doi: 10.1186/s13039-016-0266-4 27525043 PMC4981984

[26] Tran KT, Le VS, Bui HTP, Do DH, Ly HTT, Nguyen HT, et al. Genetic landscape of autism spectrum disorder in Vietnamese children. Scientific Reports. 2020;10(1):5034. doi: 10.1038/s41598-020-61695-8 32193494 PMC7081304

[27] Sharma A, Gokulchandran N, Sane H, Nivins S, Paranjape A, Badhe P. The Baseline Pattern and Age-related Developmental Metabolic Changes in the Brain of Children with Autism as Measured on Positron Emission Tomography/Computed Tomography Scan. World journal of nuclear medicine. 2018;17(2):94–101. doi: 10.4103/wjnm.WJNM_29_17 29719483 PMC5905264

[28] Sharma A. PET—CT Scan Shows Decreased Severity of Autism after Autologous Cellular Therapy: A Case Report. Autism-Open Access. 2016;06.

[29] South ST, Lee C, Lamb AN, Higgins AW, Kearney HM. ACMG Standards and Guidelines for constitutional cytogenomic microarray analysis, including postnatal and prenatal applications: revision 2013. Genetics in medicine: official journal of the American College of Medical Genetics. 2013;15(11):901–9. doi: 10.1038/gim.2013.129 24071793

[30] Kolevzon A, Angarita B, Bush L, Wang AT, Frank Y, Yang A, et al. Phelan-McDermid syndrome: a review of the literature and practice parameters for medical assessment and monitoring. Journal of Neurodevelopmental Disorders. 2014;6(1):39. doi: 10.1186/1866-1955-6-39 25784960 PMC4362650

[31] Gong X, Jiang Y-w, Zhang X, An Y, Zhang J, Wu Y, et al. High Proportion of 22q13 Deletions and SHANK3 Mutations in Chinese Patients with Intellectual Disability. PLoS ONE. 2012;7(4):e34739. doi: 10.1371/journal.pone.0034739 22509352 PMC3324537

[32] Guilmatre A, Huguet G, Delorme R, Bourgeron T. The emerging role of SHANK genes in neuropsychiatric disorders. Developmental neurobiology. 2014;74(2):113–22. doi: 10.1002/dneu.22128 24124131

[33] Phelan MC, Rogers RC, Saul RA, Stapleton GA, Sweet K, McDermid H, et al. 22q13 deletion syndrome. American journal of medical genetics. 2001;101(2):91–9. doi: 10.1002/1096-8628(20010615)101:2<91::aid-ajmg1340>3.0.co;2-c 11391650

[34] Bonaglia MC, Giorda R, Beri S, De Agostini C, Novara F, Fichera M, et al. Molecular mechanisms generating and stabilizing terminal 22q13 deletions in 44 subjects with Phelan/McDermid syndrome. PLoS Genet. 2011;7(7):e1002173. doi: 10.1371/journal.pgen.1002173 21779178 PMC3136441

[35] Leblond CS, Nava C, Polge A, Gauthier J, Huguet G, Lumbroso S, et al. Meta-analysis of SHANK Mutations in Autism Spectrum Disorders: a gradient of severity in cognitive impairments. PLoS Genet. 2014;10(9):e1004580. doi: 10.1371/journal.pgen.1004580 25188300 PMC4154644

[36] Jiang YH, Ehlers MD. Modeling autism by SHANK gene mutations in mice. Neuron. 2013;78(1):8–27. doi: 10.1016/j.neuron.2013.03.016 23583105 PMC3659167

[37] Berkel S, Marshall CR, Weiss B, Howe J, Roeth R, Moog U, et al. Mutations in the SHANK2 synaptic scaffolding gene in autism spectrum disorder and mental retardation. Nature genetics. 2010;42(6):489–91. doi: 10.1038/ng.589 20473310

[38] Pinto D, Pagnamenta AT, Klei L, Anney R, Merico D, Regan R, et al. Functional impact of global rare copy number variation in autism spectrum disorders. Nature. 2010;466(7304):368–72. doi: 10.1038/nature09146 20531469 PMC3021798

[39] Wischmeijer A, Magini P, Giorda R, Gnoli M, Ciccone R, Cecconi L, et al. Olfactory Receptor-Related Duplicons Mediate a Microdeletion at 11q13.2q13.4 Associated with a Syndromic Phenotype. Molecular syndromology. 2011;1(4):176–84. doi: 10.1159/000322054 21373257 PMC3042121

[40] Leblond CS, Heinrich J, Delorme R, Proepper C, Betancur C, Huguet G, et al. Genetic and functional analyses of SHANK2 mutations suggest a multiple hit model of autism spectrum disorders. PLoS Genet. 2012;8(2):e1002521. doi: 10.1371/journal.pgen.1002521 22346768 PMC3276563

[41] Chilian B, Abdollahpour H, Bierhals T, Haltrich I, Fekete G, Nagel I, et al. Dysfunction of SHANK2 and CHRNA7 in a patient with intellectual disability and language impairment supports genetic epistasis of the two loci. Clinical genetics. 2013;84(6):560–5. doi: 10.1111/cge.12105 23350639

[42] Schluth-Bolard C, Labalme A, Cordier MP, Till M, Nadeau G, Tevissen H, et al. Breakpoint mapping by next generation sequencing reveals causative gene disruption in patients carrying apparently balanced chromosome rearrangements with intellectual deficiency and/or congenital malformations. Journal of medical genetics. 2013;50(3):144–50. doi: 10.1136/jmedgenet-2012-101351 23315544

[43] Nguyen Thanh L, Nguyen HP. Outcomes of bone marrow mononuclear cell transplantation combined with interventional education for autism spectrum disorder. 2021;10(1):14–26. 10.1002/sctm.20-0102 PMC7780798 32902182

