# Supplementary material for: De novo copy number variations in candidate genomic regions in patients of severe autism spectrum disorder in Vietnam
Source: PLoS One. 2024 Mar 7;19(3):e0290936. doi: 10.1371/journal.pone.0290936 (PMC10919600; doi:10.1371/journal.pone.0290936)
Supplement: S1 File — (DOCX) [file pone.0290936.s001.docx]

## S1 Table: Clinical details of all 100 ASD patients

| **Proband/ Gender** | **DOB** | **Diagnosis** | | | | | **Clinical features** |
| --- | --- | --- | --- | --- | --- | --- | --- |
|  |  | Age when diagnosed (month) | | DSM-V level | ADOS score | CARS score | Delivery (weight at birth)/ Milestones & other presentations |
|  |  | First ime | Enrolling |  |  |  |  |
| ASD002/M | 2007 | 36 | 108 | 1-2 | 22/12 | 41 | C-section (3.3kg)/Walking at 12 m/o |
| ASD003/M | 1999 | 30 | 209 | 3 | 21/12 | 50 | Vaginal |
| ASD004/M | 2010 | 36 | 89 | 3 | 20/12 | 50 | C-section (2.8kg)/IVF twins, sibling with ASD, walking at 14 m/o |
| ASD005/M | 2013 | 36 | 45 | 3 | 24/12 | 50 | C-section (3.8kg), mother was 42 y/o when delivered/Walking at 24 m/o |
| ASD006/F | 2011 | 18 | 78 | 2-3 | 21/12 | 47 | C-section (3.4kg)/Walking and babbling at 13 m/o, language and intellectual regression after 18 m/o |
| ASD008/F | 2013 | 30 | 53 | 2 | 17/12 | 37 | Vaginal (3.5kg)/Walking at 16 m/o, constipation |
| ASD009/M | 2010 | 30 | 88 | 3 | 23/12 | 51 | C-section (3.5kg)/ID |
| ASD010/M | 2009 | 36 | 93 | 2 | 18/12 | 35 | Vaginal (3.2kg)/Walking and babbling at 12 m/o, language regression after 2.5 y/o, constipation |
| ASD011/M | 2011 | 24 | 74 | 3 | 21/12 | 47 | Vaginal (3.6kg)/Babbling at 7-8 m/o, walking at 10 m/o, language regression after 18 m/o |
| ASD012/M | 2003 | 18 | 161 | 2 | 20/12 | 43 | C-section (3.2kg)/Walking at 12 m/o, speech delay |
| ASD013/M | 2012 | 24 | 60 | 3 | 21/12 | 51 | C-section (3.2kg)/Babbling at 10 m/o, walking at 13.5 m/o, language regression after 14-15 m/o |
| ASD014/M | 2004 | n/a | 148 | n/a | n/a | n/a | Vaginal (3.5kg)/ |
| ASD015/M | 2012 | 24 | 60 | 3 | 22/12 | 53 | Vaginal (1.7kg)/Preterm birth at 7th months of gestation, walking at age of 3, ID, DD |
| ASD016/M | 2011 | 22 | 67 | 3 | 21/12 | 48 | Preterm birth at 33 weeks of gestation (1.9kg), GI problems |
| ASD017/F | 2006 | 18 | 126 | n/a | n/a | 53 | Vaginal (3.2kg)/Walking at 12 m/o |
| ASD018/M | 2003 | 18 | 160 | 3 | 21/12 | 49 | Vaginal (3.8kg) |
| ASD019/M | 2012 | 24 | 49 | 2 | 20/12 | 37 | C-section (3.6kg)/Walking at 12 m/o, GI problems |
| ASD020/M | 2013 | 18 | 38 | 3 | 21/12 | 46 | Vaginal (3.7kg)/walking and babbling at 13 m/o |
| ASD021/M | 2011 | 24 | 67 | 3 | 22/12 | 50 | C-section (3.1kg)/Walking at 22 m/o |
| ASD022/M | 2011 | 18 | 72 | 3 | 20/12 | 55 | C-section (3.2kg)/Walking at 15 m/o, speech delay |
| ASD023/M | 2012 | 27 | 57 | 2-3 | 18/12 | 44 | Vaginal (3.4kg) at 41 weeks of gestation/Walking at 18 m/o |
| ASD024/F | 2011 | 21 | 75 | 3 | 20/12 | 55 | Vaginal preterm birth at 37 weeks of gestation (2.2kg)/Walking at 14 m/o |
| ASD025/F | 2011 | 24 | 61 | 3 | 17/12 | 46 | Vaginal (2.8kg)/Flat feet, motor delay, ID |
| ASD026/M | 2010 | 30 | 79 | 3 | 22/12 | 45 | Vaginal (3.2kg)/Walking at 14 m/o |
| ASD027/M | 2009 | 22 | 92 | 3 | 22/12 | 47 | C-section (2.4kg)/Monozygotic twins, the sibling with ASD, walking at 15 m/o and babbling at 16 m/o, language and social regression |
| ASD028/M | 2007 | 38 | 113 | 3 | 20/12 | 42 | Vaginal (3.3kg), mother was 37 y/o/Language regression after 14 m/o |
| ASD029/M | 2012 | 48 | 57 | 3 | 23/12 | 49 | C-section (3.2kg)/Walking at 13-14 m/o |
| ASD030/F | 2010 | 22 | 79 | 2 | 22/12 | 37 | C-section (3.8kg)/Walking and babbling at 12 m/o; Language regression after 18 m/o |
| ASD031/M | 2011 | 20 | 75 | 3 | 22/12 | 55 | Vaginal (3.8kg)/Walking at 12 m/o |
| ASD032/M | 2010 | 14 | 85 | 2 | 17/10 | 42 | Vaginal (3.8kg)/Walking at 13 m/o, EP, constipation |
| ASD033/M | 2010 | 24 | 76 | 2 | 23/13 | 45 | C-section (3.5kg)/Babbling at 10 m/o, walking at 12 m/o, language regression after 12 m/o |
| ASD034/M | 2011 | 21 | 77 | 3 | 18/12 | 50 | Vaginal (3.3kg)/Babbling at 9 m/o, walking at 14 m/o, language regression after 17 m/o |
| ASD035/M | 2009 | 36 | 99 | 3 | 20/12 | 45 | Forceps delivery/Walking at 17 m/o, babbling at 24 m/o |
| ASD036/F | 2009 | 18 | 57 | 3 | 20/12 | 49 | Vaginal (3.5kg)/DD |
| ASD037/M | 2012 | 24 | 52 | 3 | 52 | 51 | Vaginal (3.2kg), preterm birth at 32 weeks of gestation/ Babbling at 10 m/o and walking at 16 m/o |
| ASD038/M | 2010 | 24 | 48 | n/a | 22/12 | 48 | C-section (2.6kg)/Missed rolling and clawing, walking at 2 y/o; CP, ID |
| ASD039/M | 2010 | 18 | 81 | 3 | 22/12 | 53 | Vaginal (3.5kg)/Babbling at 12 m/o, walking at 15 m/o |
| ASD040/M | 2011 | 20 | 70 | 3 | 22/12 | 52 | Vaginal (3.2kg)/ Walking and babbling at 12 m/o, language regression after 18 m/o |
| ASD041/M | 2003 | 36 | 169 | 3 | 21/12 | 49 | C-section (3.6kg)/Walking at 17-18 m/o, speech delay |
| ASD042/M | 2011 | 36 | 71 | 3 | 22/12 | 48 | Vaginal (3kg)/Walking at 18 m/o, speech delay |
| ASD043/M | 2003 | 36 | 166 | 2 | 22/12 | 44 | Vaginal (3.3kg)/Walking at 15 m/o, sensation disorders |
| ASD044/M | 2012 | 24 | 60 | 3 | 20/12 | 53 | C-section (3.2kg)/Missed crawling, walking at 21 m/o, language regression after 7-8 m/o, EP at 12 m/o |
| ASD045/M | 2011 | 27 | 64 | 3 | 24/12 | 50 | Vaginal (3.4kg)/Walking at 14 m/o, speech delay |
| ASD046/M | 2006 | 24 | 152 | 3 | 21/12 | 46 | Vaginal (3.2kg)/Babbling at 18-20 m/o, walking at 3 y/o, language regression after 2 y/o, motor delay |
| ASD047/M | 2009 | 18 | 90 | 3 | 22/12 | 50 | C-section (2.9kg)/Babbling at 7-8 m/o, walking at 28 m/o, ID, disrupted sleep |
| ASD048/M | 2007 | 18 | 119 | 2 | 20/12 | 40 | Vaginal (3.7kg)/Walking at 13 m/o, sensation disorders |
| ASD049/M | 2007 | 24 | 115 | 3 | 21/12 | 46 | C-section (3.7kg)/Walking at 12-13 m/o, language regression after 18 m/o |
| ASD050/M | 2011 | 36 | 66 | 3 | 22/12 | 52 | C-section (3kg), mother was 40 y/o when delivered/Babbling at 11 m/o, walking at 17 m/o |
| ASD051/F | 2007 | 24 | 112 | 1-2 | n/a | 35 | Vaginal (2.6kg)/Walking at 12 m/o, babbling at 24 m/o |
| ASD052/M | 2014 | 18 | 30 | 2 | 20/12 | n/a | Vaginal (3.7kg)/Walking at 13 m/o, talking at 30 m/o, DD |
| ASD053/M | 2010 | 30 | 86 | 3 | 21/12 | 46 | Vaginal (3.5kg)/Walking and babbling at 11 m/o |
| ASD054/M | 2011 | 24 | 76 | 3 | 16/12 | 50 | C-section (2.7kg), mother was infected with rubella at 4th months of gestation/Walking at 18 m/o, babbling at 36 m/o, sleep deprivation, ID |
| ASD056/M | 2007 | 36 | 119 | 2 | 17/12 | 40 | Vaginal (3.4kg)/Walking at 17-18 m/o and babbling at 24 m/o, language regression after 24 m/o |
| ASD057/M | 2011 | 36 | 70 | 3 | 20/12 | 48 | C-section (2.5kg)/Walking at12 m/o, speech delay |
| ASD058/M | 2012 | 24 | 59 | 3 | 20/12 | 50 | Vaginal (4.2kg) |
| ASD059/M | 2010 | 22 | 80 | 2-3 | 21/12 | 46 | C-section (3kg)/Sensation disorders |
| ASD060/M | 2012 | 16 | 54 | 2 | 15/12 | 38 | Vaginal (3.4kg)/Walking at 12 m/o, talking at 24 m/o |
| ASD061/M | 2013 | 18 | 49 | 3 | 21/12 | 45 | C-section (3.4kg)/Walking at 14 m/o, babbling at 9 m/o, language regression after 12 m/o |
| ASD062/M | 2011 | 28 | 74 | 3 | 18/12 | 46 | Vaginal (3.3kg)/2nd child, older brother presented speech delay, walking at 12 m/o, sleep disorder |
| ASD063/M | 2012 | 36 | 56 | 3 | 20/12 | 45 | C-section (4.1kg)/Walking at 15 m/o |
| ASD064/M | 2007 | 24 | 117 | 2 | 15/12 | 40 | C-section (3.4kg)/Walking at 14-15 m/o, speech delay |
| ASD065/F | 2010 | 83 | 83 | 2-3 | 22/12 | 43 | C-section (3.35kg)/Older brother with CP, walking and babbling at 12 m/o, language regression after 12 m/o, EP at 8 m/o, sensation disorders, DD |
| ASD066/M | 2012 | 24 | 52 | 3 | 21/12 | 50 | C-section (2.9kg), mother was 43 y/o when delivered/Walking at 16 m/o |
| ASD067/M | 2009 | 18 | 98 | 3 | 22/12 | 46 | Vaginal (3.2kg)/Walking at 14 m/o, babbling at 20 m/o, sleep disorder |
| ASD068/F | 2013 | 18 | 44 | 3 | 20/12 | 54 | Vaginal (4.2kg)/Walking at 12 m/o, EP |
| ASD069/M | 2011 | 24 | 73 | 3 | 22/12 | 49 | Vaginal (3.1kg), mother was 39 y/o when delivered/Walking at 15 m/o, talking at 48 m/o |
| ASD070/M | 2013 | 18 | 42 | 3 | 20/12 | 49 | Vaginal (2.9kg)/Walking and babbling at 12 m/o, language regression after 16 m/o |
| ASD071/M | 2012 | 36 | 59 | 3 | 20/12 | 53 | C-section (3kg), mother was under depression treatment during pregnancy/Walking at 22 m/o, babbling at 12 m/o, language regression after 18 m/o |
| ASD072/M | 2012 | 24 | 59 | 3 | 20/12 | 56 | Vaginal (1.3kg), preterm birth at 7th month of gestation/Older brother with speech delay; Walking at 15 m/o, babbling at 18 m/o, asthma |
| ASD073/F | 2012 | 24 | 55 | 3 | 20/12 | 50 | C-section (3.9kg)/Walking at 9 m/o |
| ASD074/M | 2009 | 21 | 95 | 2-3 | 21/12 | 39 | Vaginal (3.4kg) at 37 weeks of gestation/ Visual defect, walking at 18 m/o, talking at 12 m/o, sleep deprivation |
| ASD075/M | 2012 | 24 | 59 | 3 | 19/12 | 51 | C-section (3.1kg), birth asphyxia/Older brother with speech delay and ID |
| ASD076/M | 2011 | 24 | 70 | 3 | 20/12 | 54 | C-section (3.7kg)/Walking at 14 m/o, babbling at 12 m/o, language regression |
| ASD077/M | 2011 | 30 | 71 | 2-3 | 18/12 | 42 | C-section at 41 weeks of gestation (3.6kg)/Walking at 13 m/o, babbling at 24 m/o |
| ASD078/M | 2014 | 18 | 36 | 3 | 16/12 | 45 | Emergency C-section at 38 weeks of gestation (3.5kg) due to intrauterine growth restriction |
| ASD079/M | 2011 | 20 | 70 | 3 | 24/12 | 51 | C-section (3.6kg)/ Walking at 12 m/o |
| ASD080/F | 2012 | 48 | 66 | 3 | 21/12 | 50 | Vaginal/Walking at 2 y/o, disrupted sleep |
| ASD081/M | 2013 | 19 | 54 | 3 | 20/12 | 49 | C-section at 37 weeks of gestation (3.7kg)/Walking at 13 m/o, talking at 13 m/o, language regression after 17-18 m/o |
| ASD082/F | 2012 | 24 | 62 | 3 | n/a | 45 | C-section (2.9kg)/ Congenital foot deformity, walking at 19-20 m/o |
| ASD083/M | 2011 | 26 | 50 | 2-3 | 20/12 | 46 | C-section (3.2kg)/ Walking and babbling at 9 m/o |
| ASD084/M | 2013 | 24 | 53 | 3 | 22/12 | 46 | Vaginal (3.3kg)/Walking at 13 m/o, babbling at 18 m/o, language delay after 18 m/o |
| ASD085/M | 2013 | 18 | 42 | 3 | 20/12 | 44 | Vaginal at 35 weeks of gestation (2.4kg)/Walking at 12 m/o |
| ASD086M | 2012 | 17 | 57 | 2-3 | 21/12 | 45 | C-section (4kg)/Walking at 12 m/o, speech delay |
| ASD087/M | 2010 | 36 | 88 | 3 | 23/12 | 44 | Vaginal (3.3kg) |
| ASD088/M | 2014 | 39 | 41.5 | 2-3 | 20/12 | 39 | C-section at 36 weeks of gestation (3.6kg)/Walking at 13 m/o, babbling at 9 m/o, often constipation |
| ASD089/M | 2012 | 36 | 62 | 3 | 20/12 | 54 | Vaginal (3.3kg)/Walking at 24 m/o, ID, decrease perception of pain |
| ASD090/M | 2010 | 24 | 80 | 3 | 18/12 | 49 | Vaginal (3.5kg)/Walking at 10 m/o |
| ASD091/F | 2009 | 21 | 99 | 3 | 22/12 | 51 | Vaginal preterm birth at 36 weeks of gestation (3.8kg), ID |
| ASD092/M | 2012 | 24 | 63 | 3 | 19/12 | 53 | C-section (3.9kg)/ Walking at 14 m/o, babbling at 10 m/o, language regression |
| ASD093/M | 2008 | 22 | 109 | 3 | 19/12 | 45 | C-section (3kg)/ Walking at 17 m/o, babbling at 18-19 m/o |
| ASD094/M | 2009 | 30 | 95 | 1-2 | 15/12 | 41 | Vaginal, no concern at birth |
| ASD095/M | 2012 | 18 | 62 | 3 | 22/12 | 53 | Vaginal (3kg)/Chronic constipation, malnutrition |
| ASD096/M | 2009 | 26 | 87 | 3 | 22/12 | 48 | C-section (3.6kg)/Normal motor milestones (crawling, walking) |
| ASD097/M | 2004 | 24 | 154 | 3 | 22/12 | 50 | Vaginal (3.2kg), mother was 35 y/o/Walking at 24 m/o, speech delay, sleep deprivation, ID |
| ASD098/F | 2009 | 18 | 88 | n/a | n/a | 41 | Vaginal (3.2kg), full term/ Rolling at 5 m/o, walking at 15 m/o, babbling at 14 m/o, language and social interaction regressed after 16 m/o |
| ASD099/M | 2008 | 24 | 66 | 3 | 21/12 | 48 | Vaginal at 38 weeks of gestation (3.7kg)/Walking at 14 m/o, babbling at 12 m/o, sleep disorder, Chiari I, ID |
| ASD100/F | 2008 | 36 | 95 | n/a | n/a | 48 | C-section (3.4kg), mother was 36 y/o/Walking at 12 m/o |
| ASD101/M | 2011 | 24 | 68 | 3 | n/a | 48 | Vaginal, birth asphyxia/Walking at 30 m/o, ID |
| ASD102/M | 2014 | 19 | 35 | 2-3 | 22/12 | 44 | C-section (2.6kg) at 37 weeks of gestation/Walking at 19 m/o, speech delay |
| ASD103/M | 2009 | 36 | 96 | 2-3 | 22/12 | 43 | C-section (3.1kg)/Walking at 12 m/o, speech delay |

**S2 Table: Details of clinical features**

**Social interaction, language, repetitive and restricted activities and interests are clinical criteria listed in 100 patients divided in male and female**

| **Domain** | **Male**  **N** | **Female**  **N** |
| --- | --- | --- |
| ***Social interaction*** | | |
| No social interaction | 24 | 3 |
| Have social interaction | 59 | 14 |
| ***Eye contact*** | | |
| No eye contact | 16 | 2 |
| Little and normal eye contact | 67 | 15 |
| ***Expression of feeling to parent*** | | |
| Showing affection | 66 | 14 |
| No showing affection | 17 | 3 |
| ***Expressive language*** | | |
| No language | 35 | 6 |
| Single word | 36 | 6 |
| Sentence | 12 | 5 |
| *Stereotypic/****Repetitive Behaviors*** | | |
| Much repetitive behavior | 54 | 5 |
| Less repetitive behavior | 29 | 12 |
| No repetitive behavior | 0 | 0 |
| ***Restricted interests*** | | |
| Much restricted interest | 59 | 5 |
| Less restricted interest | 33 | 12 |
| No restricted interest | 1 | 0 |
| ***Hyperactivity*** | | |
| Have hyperactivity | 53 | 12 |
| No hyperactivity | 20 | 5 |
| ***Self-injurious behavior*** | | |
| Have self-injurious behavior | 12 | 3 |
| No self-injurious behavior | 71 | 14 |
| ***Sensory Impairments*** | | |
| Have sensory abnormalities | 69 | 9 |
| No sensory abnormalities | 14 | 8 |
| ***Picky eating behaviours*** | | |
| Have picky eating behavior | 38 | 5 |
| No picky eating behavior | 45 | 12 |
| ***Sleep problems*** | | |
| Have sleep problems | 16 | 2 |
| No sleep problems | 67 | 15 |
| ***Self-feeding*** | | |
| Unable to self-feed | 11 | 3 |
| Need support to self-feed | 35 | 3 |
| Able to self-feed | 37 | 11 |
| ***Toileting skills*** | | |
| Unable to go to toilet | 23 | 4 |
| Need support to go to toilet | 27 | 2 |
| Able to go to toilet | 33 | 11 |
| ***Learning ability*** | | |
| Unable to integrate into school | 68 | 11 |
| Need support to integrate into school (with help from teachers) | 14 | 5 |
| Go to school normally | 1 | 1 |
| ***Gut issue*** | | |
| Have gut issue | 4 | 0 |
| Normal | 79 | 17 |

## S3 Table: Brain imaging information in Probands

| **Proband/ Gender** | **Brain imaging** | |
| --- | --- | --- |
|  | FDG-PET/CT  (FDG hypometabolism) | MRI |
| ASD002/M | n/a | NAD |
| ASD003/M | n/a | n/a |
| ASD004/M | Parietal lobe, central sulcus, hippocampus, anterior cingulate gyrus | n/a |
| ASD005/M | n/a | n/a |
| ASD006/F | Temporal lobe, hippocampus, right parietal lobe | n/a |
| ASD008/F | n/a | n/a |
| ASD009/M | n/a | n/a |
| ASD010/M | n/a | n/a |
| ASD011/M | Hippocampus, central sulcus, parietal lobe, anterior cingulate gyrus | n/a |
| ASD012/M | n/a | n/a |
| ASD013/M | Temporal lobe, hippocampus, cerebellum | n/a |
| ASD014/M | n/a | n/a |
| ASD015/M | n/a | ABN |
| ASD016/M | n/a | n/a |
| ASD017/F | n/a | ABN |
| ASD018/M | n/a | n/a |
| ASD019/M | n/a | n/a |
| ASD020/M | n/a | n/a |
| ASD021/M | Anterior cingulate gyrus | n/a |
| ASD022/M | Temporal lobe, bilateral hippocampus, prefrontal cortex | NAD |
| ASD023/M | n/a | n/a |
| ASD024/F | Parietal lobe, central sulcus, temporal lobe, hippocampus, anterior cingulate gyrus | ABN |
| ASD025/F | n/a | ABN |
| ASD026/M | n/a | n/a |
| ASD027/M | n/a | n/a |
| ASD028/M | n/a | NAD |
| ASD029/M | n/a | ABN |
| ASD030/F | n/a | NAD |
| ASD031/M | Parietal lobe, central sulcus, temporal lobe, hippocampus, anterior cingulate gyrus | n/a |
| ASD032/M | n/a | n/a |
| ASD033/M | n/a | n/a |
| ASD034/M | Hippocampus, anterior cingulate gyrus | NAD |
| ASD035/M | n/a | ABN |
| ASD036/F | n/a | NAD |
| ASD037/M | n/a | n/a |
| ASD038/M | n/a | NAD |
| ASD039/M | n/a | NAD |
| ASD040/M | Parietal lobe, anterior cingulate gyrus, hippocampus | n/a |
| ASD041/M | n/a | n/a |
| ASD042/M | n/a | n/a |
| ASD043/M | n/a | n/a |
| ASD044/M | Parietal lobe, central sulcus, temporal lobe, hippocampus, anterior cingulate gyrus | NAD |
| ASD045/M | Temporal lobe, hippocampus, anterior cingulate gyrus | ABN |
| ASD046/M | n/a | NAD |
| ASD047/M | n/a | NAD |
| ASD048/M | n/a | n/a |
| ASD049/M | n/a | n/a |
| ASD050/M | n/a | n/a |
| ASD051/F | n/a | ABN |
| ASD052/M | n/a | n/a |
| ASD053/M | n/a | n/a |
| ASD054/M | n/a | ABN |
| ASD056/M | n/a | NAD |
| ASD057/M | n/a | n/a |
| ASD058/M | n/a | n/a |
| ASD059/M | n/a | NAD |
| ASD060/M | n/a | n/a |
| ASD061/M | n/a | n/a |
| ASD062/M | Parietal lobe, central sulcus, hippocampus, anterior cingulate gyrus | n/a |
| ASD063/M | Hippocampus, frontal lobe | NAD |
| ASD064/M | n/a | n/a |
| ASD065/F | n/a | n/a |
| ASD066/M | n/a | n/a |
| ASD067/M | n/a | n/a |
| ASD068/F | n/a | NAD |
| ASD069/M | Hippocampus, anterior cingulate gyrus | NAD |
| ASD070/M | Central sulcus, hippocampus | n/a |
| ASD071/M | Central sulcus, hippocampus, anterior cingulate gyrus | NAD |
| ASD072/M | Parietal lobe, hippocampus, anterior cingulate gyrus | NAD |
| ASD073/F | Temporal lobe, hippocampus, anterior cingulate gyrus | n/a |
| ASD074/M | n/a | ABN |
| ASD075/M | Hippocampus, parietal lobe, anterior cingulate gyrus | n/a |
| ASD076/M | Hippocampus, frontal lobe | n/a |
| ASD077/M | Hippocampus, anterior cingulate gyrus | n/a |
| ASD078/M | n/a | n/a |
| ASD079/M | Parietal lobe, hippocampus, anterior cingulate gyrus | n/a |
| ASD080/F | Frontal lobe, anterior cingulate gyrus, hippocampus | n/a |
| ASD081/M | Hippocampus, anterior cingulate gyrus | n/a |
| ASD082/F | n/a | n/a |
| ASD083/M | n/a | n/a |
| ASD084/M | n/a | n/a |
| ASD085/M | n/a | NAD |
| ASD086M | n/a | n/a |
| ASD087/M | n/a | ABN |
| ASD088/M | n/a | n/a |
| ASD089/M | n/a | ABN |
| ASD090/M | Anterior cingulate gyrus, hippocampus | n/a |
| ASD091/F | n/a | NAD |
| ASD092/M | n/a | ABN |
| ASD093/M | n/a | NAD |
| ASD094/M | n/a | n/a |
| ASD095/M | Central sulcus, hippocampus, anterior cingulate gyrus | n/a |
| ASD096/M | n/a | NAD |
| ASD097/M | n/a | NAD |
| ASD098/F | n/a | ABN |
| ASD099/M | n/a | ABN |
| ASD100/F | n/a | NAD |
| ASD101/M | Hippocampus, anterior cingulate gyrus | n/a |
| ASD102/M | Hippocampus, anterior cingulate gyrus | n/a |
| ASD103/M | n/a | NAD |

## S4 Table: Details of 118 unique CNVs

| No | Chr | Position | Start | End | Probes | DLRS | P value | Genes |
| --- | --- | --- | --- | --- | --- | --- | --- | --- |
| 1 | chr1 | p22.3 | 87099791 | 87105798 | 3 | -1.01571 | 9.49E-12 | *LOC105378828, CLCA3P* |
| 2 | chr1 | p36.13 | 16927124 | 17231817 | 12 | 0.492793 | 6.06E-16 | *NBPF1, CROCCP2, MST1P2, FAM231AP, FAM231A, FAM231C, MIR3675, ESPNP, MST1L, LOC102724562, LOC440570, LOC105376805* |
| 3 | chr1 | p32.1 | 59842022 | 59878289 | 8 | -0.52676 | 9.62E-10 | *FGGY* |
| 4 | chr1 | p22.1 | 93002721 | 93031671 | 6 | -1.07901 | 1.16E-24 | *EVI5* |
| 5 | chr1 | p36.11 | 25675212 | 25688208 | 4 | 0.98616 | 1.02E-20 | *TMEM50A* |
| 6 | chr1 | p36.22 | 10505050 | 10516509 | 4 | 0.680744 | 1.63E-10 | *CENPS-CORT, CORT* |
| 7 | chr1 | q21.3 | 152766564 | 152773898 | 3 | -0.88721 | 7.34E-12 | *LCE1D* |
| 8 | chr1 | q31.3 | 196705001 | 196781644 | 6 | -0.60548 | 2.27E-10 | *CFH, CFHR3* |
| 9 | chr1 | q44 | 248601802 | 248646776 | 7 | 0.520891 | 1.88E-10 | *OR2T7, OR2T2, OR2T3* |
| 10 | chr1 | p36.33 | 1585691 | 1613809 | 9 | 0.483988 | 3.05E-12 | *CDK11B, SLC35E2B* |
| 11 | chr1 | p36.13 | 17207777 | 17276064 | 9 | -0.71563 | 1.45E-20 | *LOC105376805, CROCC* |
| 12 | chr1 | q42.13 | 228783832 | 228788356 | 3 | -0.96754 | 3.39E-14 | *RHOU, DUSP5P1* |
| 13 | chr1 | q24.2 | 167638467 | 167646427 | 3 | -0.76556 | 6.47E-10 | *RCSD1* |
| 14 | chr1 | p36.23 | 8672082 | 8688845 | 3 | -1.02107 | 7.65E-15 | *RERE* |
| 15 | chr1 | p13.2 | 111832138 | 111835175 | 3 | -0.95271 | 8.55E-14 | *CHIA* |
| 16 | chr1 | q21.1 | 144986366 | 145076206 | 21 | -0.3907 | 4.07E-18 | *NBPF20, NBPF19, NBPF9, LOC100996724, PDE4DIP* |
| 17 | chr1 | q42.2 | 231535636 | 231542955 | 3 | -0.77262 | 4.08E-10 | *EGLN1* |
| 18 | chr2 | q11.2 | 100,758,125 | 100,765,082 | 3 | -0.80014 | 1.36E-10 | *AFF3* |
| 19 | chr2 | q37.3 | 242,750,401 | 242,758,759 | 3 | 0.845233 | 1.10E-11 | *NEU4* |
| 20 | chr2 | p13.3 | 71,337,169 | 71,354,407 | 4 | -0.66806 | 5.02E-10 | *MCEE* |
| 21 | chr3 | p21.31 | 48,580,689 | 48,680,593 | 20 | 0.25401 | 1.62E-10 | *PFKFB4, MIR6823, UCN2, COL7A1, MIR711, UQCRC1, SNORA94, TMEM89, SLC26A6, MIR6824, CELSR3* |
| 22 | chr3 | q25.32 | 157,850,581 | 157,870,391 | 5 | -0.61068 | 9.18E-10 | *RSRC1* |
| 23 | chr4 | q31.21 | 144,805,113 | 145,057,804 | 18 | 0.439419 | 1.12E-15 | *GYPE, LOC101927636, GYPB, GYPA* |
| 24 | chr4 | q32.3 | 166,334,022 | 166,351,395 | 5 | -0.62225 | 2.85E-11 | *CPE* |
| 25 | chr4 | q32.2 | 162,474,015 | 162,488,007 | 4 | 0.689832 | 7.47E-10 | *FSTL5* |
| 26 | chr4 | p16.1 | 6,401,138 | 6,413,973 | 4 | -0.5961 | 2.48E-10 | *PPP2R2C* |
| 27 | chr4 | q31.21 | 144,986,413 | 145,032,578 | 6 | 0.565563 | 2.85E-10 | *GYPA* |
| 28 | chr4 | q22.2 | 93,919,081 | 93,969,117 | 10 | -0.70495 | 6.01E-24 | *GRID2* |
| 29 | chr4 | q34.1 - q35.2 | 175,897,368 | 190,896,674 | 1847 | -0.78104 | 4.900e-324 | *ADAM29, GPM6A, LOC101928590, WDR17, SPATA4, ASB5, SPCS3, VEGFC, LINC02509, NEIL3, AGA, LINC01098, LINC01099, LINC00290, LINC02500, TEMN3-AS1, LOC90768, MIR1305, TENM3, DCTD, FAM92A1P2, WWC2-AS2, WWC2, WWC2-AS1, CLDN22, CLDN24, CDKN2AIP, LOC389247, ING2, RWDD4, TRAPPC11, STOX2, ENPP6, LINC02363, LINC02362, IRF2, LINC02427, LINC02365, CASP3, PRIMPOL, CENPU, ACSL1, SLED1, MIR3945HG, MIR3945, LINC01093, MIR4455, HELT, LINC02436, SLC25A4, CFAP97, SNX25, LRP2BP, ANKRD37, UFSP2, C4orf47, CCDC110, LOC105377590, PDLIM3, SORBS2, TLR3, FAM149A, FLJ38576, CYP4V2, KLKB1, F11, F11-AS1, MTNR1A, FAT1, LINC02374, LOC339975, LINC02514, LINC02492, ZFP42, TRIML2, TRIML1, LINC01060, LINC02508, LINC01262, LOC105379514, FRG1-DT, LINC01596, FRG1* |
| 30 | chr5 | p15.33 | 685,256 | 777,000 | 5 | 0.665994 | 8.79E-10 | *TPPP, ZDHHC11B* |
| 31 | chr5 | q11.2 | 51,335,134 | 51,426,885 | 5 | 0.827911 | 1.73E-10 | *LINC02118* |
| 32 | chr5 | p15.33 - p15.32 | 1,910,175 | 5,391,389 | 451 | 0.305105 | 5.55E-28 | *LOC100506858, LSINCT5, IRX2, C5orf38, LOC105374620, LINC01377, LINC01019, LINC02162, LINC01017, IRX1, LINC02114, LINC01020, LINC02121, CTD-2297D10.2, ADAMTS16* |
| 33 | chr5 | q31.3 | 140,210,459 | 140,216,357 | 3 | -0.98042 | 1.76E-11 | *PCDHA1, PCDHA2, PCDHA3, PCDHA4, PCDHA5, PCDHA6, PCDHA7* |
| 34 | chr5 | q13.2 | 68,818,173 | 70,657,747 | 8 | -0.67097 | 6.85E-15 | *OCLN, SNORD13B-2, SNORD13B-1, GTF2H2C_2, GTF2H2C, GUSBP3, LOC653080, SERF1A, SERF1B, SMN2, SMN1, SMA4, GTF2H2B, SMA5, LOC441081, GUSBP9, NAIP, GTF2H2, LOC647859, LINC02197* |
| 35 | chr5 | q14.3 | 90,077,035 | 90,083,071 | 3 | 0.836005 | 5.95E-10 | *ADGRV1* |
| 36 | chr5 | q13.2 | 68,815,067 | 68,824,041 | 3 | -0.78937 | 5.17E-10 | *OCLN* |
| 37 | chr6 | p21.31 | 35,745,231 | 35,765,416 | 4 | 0.700719 | 5.04E-10 | *CLPSL2, CLPSL1, CLPS* |
| 38 | chr6 | p25.2 | 3,719,574 | 3,727,801 | 3 | -0.86386 | 8.63E-12 | *PXDC1* |
| 39 | chr6 | q14.3 | 86,373,607 | 86,395,961 | 5 | 0.69426 | 1.90E-13 | *SNHG5, SNORD50A, SNORD50B* |
| 40 | chr6 | p22.1 | 29,913,929 | 29,945,434 | 6 | -0.55782 | 5.98E-11 | *HCG9* |
| 41 | chr7 | q35 | 143,425,418 | 143,466,132 | 5 | 0.643663 | 1.00E-10 | *TCAF2, CTAGE6* |
| 42 | chr7 | q34 | 142,340,360 | 142,497,079 | 26 | -0.44212 | 2.92E-25 | *MTRNR2L6, PRSS1, PRSS3P2* |
| 43 | chr7 | q35 | 147,531,869 | 147,551,624 | 5 | -0.51394 | 1.63E-10 | *CNTNAP2* |
| 44 | chr7 | q35 | 143,951,931 | 144,074,551 | 4 | -0.6545 | 2.44E-10 | *LOC101928605, OR2A1-AS1, OR2A7, ARHGEF34P, CTAGE4, CTAGE8, OR2A20P, OR2A9P, OR2A1, OR2A42, ARHGEF5* |
| 45 | chr7 | q22.1 | 100,333,268 | 100,341,714 | 3 | -0.80157 | 2.70E-10 | *ZAN* |
| 46 | chr7 | q35 | 143,466,073 | 143,547,906 | 3 | -0.87333 | 3.91E-10 | *TCAF2P1, LOC154761* |
| 47 | chr7 | q35 | 143,257,517 | 143,434,537 | 5 | -0.93277 | 2.14E-20 | *CTAGE15, TCAF2, TCAF2P1* |
| 48 | chr8 | p22 | 16,954,134 | 16,984,198 | 7 | 0.714118 | 3.56E-11 | *MICU3* |
| 49 | chr8 | p23.2 | 3,545,824 | 3,583,362 | 9 | 0.689289 | 3.49E-10 | *CSMD1* |
| 50 | chr8 | p23.1 | 10,403,206 | 10,416,312 | 3 | -0.8661 | 3.34E-11 | *PRSS55* |
| 51 | chr8 | p12 | 32,421,470 | 32,438,790 | 5 | -0.58294 | 1.45E-10 | *NRG1* |
| 52 | chr9 | p23 | 10,550,843 | 10,558,986 | 3 | -0.78138 | 4.83E-11 | *PTPRD* |
| 53 | chr9 | q22.31 | 96,166,255 | 96,227,148 | 10 | -0.55163 | 2.94E-10 | *FAM120AOS, FAM120A* |
| 54 | chr9 | q31.1 | 108,164,850 | 108,181,793 | 4 | 0.698271 | 1.46E-10 | *SLC44A1* |
| 55 | chr9 | q21.33 | 88,823,348 | 88,840,945 | 4 | -0.89031 | 8.98E-18 | *C9orf153* |
| 56 | chr9 | q34.3 | 139,326,377 | 139,516,668 | 38 | 0.395903 | 1.25E-17 | *INPP5E, SEC16A, C9orf163, NOTCH1, MIR4673, MIR4674, NALT1, LINC01451* |
| 57 | chr10 | q25.1 | 108,453,644 | 108,473,165 | 4 | 0.768123 | 1.88E-10 | *SORCS1* |
| 58 | chr10 | p11.21 | 37,450,623 | 37,468,455 | 3 | 0.978304 | 3.56E-11 | *ANKRD30A* |
| 59 | chr10 | p15.3 | 1,518,099 | 1,525,945 | 3 | -0.66806 | 3.93E-10 | *ADARB2* |
| 60 | chr11 | q13.2 - q13.4 | 67,856,136 | 73,062,386 | 810 | -0.86419 | 4.900e-324 | *CHKA, KMT5B, C11orf24, LRP5, PPP6R3, GAL, TESMIN, CPT1A, MRPL21, IGHMBP2, MRGPRD, MRGPRF, MRGPRF-AS1, TPCN2, MIR3164, LOC338694, MYEOV, LOC102724265, LINC01488, CCND1, ORAOV1, FGF19, FGF4, FGF3, LOC101928443, ANO1-AS2, ANO1, FADD, PPFIA1, MIR548K, CTTN, SHANK2, SHANK2-AS1, SHANK2-AS3, MIR3664, FLJ42102, DHCR7, NADSYN1, MIR6754, KRTAP5-7, KRTAP5-8, KRTAP5-9, KRTAP5-10, KRTAP5-11, FAM86C1, ALG1L9P, ZNF705E, DEFB108B, LOC100133315, DEFB131B, RNF121, IL18BP, NUMA1, LOC100128494, MIR3165, LRTOMT, LAMTOR1, ANAPC15, FOLR3, FOLR1, FOLR2, INPPL1, PHOX2A, CLPB, LINC01537, PDE2A, MIR139, ARAP1, ARAP1-AS2, STARD10, MIR4692, ATG16L2, FCHSD2, MIR4459, P2RY2, P2RY6, LOC100287837, ARHGEF17* |
| 61 | chr11 | p15.4 | 4,962,435 | 4,987,947 | 6 | 0.579683 | 1.64E-11 | *OR51A4, OR51A2* |
| 62 | chr11 | q14.3 | 89,856,654 | 89,885,908 | 6 | 0.68939 | 8.65E-11 | *NAALAD2* |
| 63 | chr11 | q22.3 | 107,655,060 | 107,673,765 | 5 | -0.59082 | 4.68E-10 | *SLC35F2* |
| 64 | chr11 | q23.3 | 117,051,017 | 117,070,410 | 5 | 0.489845 | 8.70E-10 | *SIDT2, LOC100652768, TAGLN* |
| 65 | chr11 | q25 | 132,574,785 | 132,586,756 | 4 | 0.840603 | 5.45E-10 | *OPCML* |
| 66 | chr11 | p11.12 | 50,378,743 | 51,511,461 | 11 | 0.507499 | 1.95E-15 | *LOC646813, OR4A5* |
| 67 | chr11 | p14.3 | 24,924,128 | 24,971,889 | 11 | 0.645227 | 1.05E-11 | *LUZP2* |
| 68 | chr11 | p15.4 | 4,962,435 | 4,971,528 | 3 | 0.686155 | 1.01E-09 | *OR51A4* |
| 69 | chr11 | p15.1 | 16,917,227 | 16,925,760 | 3 | -1.43093 | 1.26E-21 | *PLEKHA7* |
| 70 | chr11 | q14.1 | 78,368,953 | 78,399,909 | 7 | -0.55249 | 3.23E-11 | *TENM4* |
| 71 | chr12 | p13.2 | 10,492,679 | 10,521,371 | 6 | -0.68832 | 2.17E-13 | *LOC101928100* |
| 72 | chr13 | q12.3 | 29,749,504 | 29,759,979 | 3 | 0.838787 | 1.23E-10 | *MTUS2* |
| 73 | chr14 | q32.33 | 104,546,943 | 104,623,679 | 25 | 0.294784 | 1.19E-10 | *ASPG, MIR203A, MIR203B, KIF26A* |
| 74 | chr14 | q12 | 31,579,681 | 31,589,026 | 3 | -0.82288 | 9.63E-10 | *HECTD1* |
| 75 | chr14 | q11.2 | 22,277,272 | 23,266,159 | 197 | -0.29822 | 1.64E-87 | *LOC105370401, LINC02332, DAD1, ABHD4, OR6J1, OXA1L, SLC7A7* |
| 76 | chr15 | q11.2 | 25,430,535 | 25,499,179 | 14 | 0.505078 | 1.15E-10 | *SNHG14, SNORD115-9, SNORD115-10, SNORD115-12, SNORD115-5, SNORD115-11, SNORD115-29, SNORD115-36, SNORD115-43, SNORD115-13, SNORD115-14, SNORD115-16, SNORD115-17, SNORD115-18, SNORD115-19, SNORD115-20, SNORD115-15, SNORD115-21, SNORD115-22, PWAR4, SNORD115-23, SNORD115-24, SNORD115-25, SNORD115-26, SNORD115-27, SNORD115-28, SNORD115-30, SNORD115-31, SNORD115-32, SNORD115-33, SNORD115-34, SNORD115-35, SNORD115-37, SNORD115-38, SNORD115-39, SNORD115-40, SNORD115-41, SNORD115-42, SNORD115-44* |
| 77 | chr15 | q11.1 - q11.2 | 20,432,851 | 22,835,945 | 71 | -0.33186 | 3.18E-39 | *CHEK2P2, HERC2P3, GOLGA6L6, GOLGA8CP, NBEAP1, MIR3118-2, MIR3118-3, MIR3118-4, POTEB, POTEB2, POTEB3, NF1P2, MIR5701-1, MIR5701-2, MIR5701-3, LINC01193, LINC02203, FAM30C, LOC646214, CXADRP2, LOC101927079, OR4M2, OR4N4, OR4N3P, IGHV1OR15-1, LOC102724760, IGHV1OR15-3, LOC642131, MIR1268A, REREP3, MIR4509-1, MIR4509-2, MIR4509-3, GOLGA8DP, GOLGA6L1, GOLGA6L22, TUBGCP5* |
| 78 | chr15 | q24.3 | 76,883,703 | 76,893,443 | 3 | 0.810683 | 2.22E-10 | *SCAPER* |
| 79 | chr15 | q11.1 - q13.3 | 20,102,541 | 32,445,252 | 1318 | 0.92734 | 4.900e-324 | *CHEK2P2, HERC2P3, GOLGA6L6, GOLGA8CP, NBEAP1, MIR3118-2, MIR3118-3, MIR3118-4, POTEB, POTEB2, POTEB3, NF1P2, MIR5701-1, MIR5701-2, MIR5701-3, LINC01193, LINC02203, FAM30C, LOC646214, CXADRP2, LOC101927079, OR4M2, OR4N4, OR4N3P, IGHV1OR15-1, LOC102724760, IGHV1OR15-3, LOC642131, MIR1268A, REREP3, MIR4509-1, MIR4509-2, MIR4509-3, GOLGA8DP, GOLGA6L1, GOLGA6L22, TUBGCP5, CYFIP1, NIPA2, NIPA1, LOC283683, WHAMMP3, GOLGA8IP, HERC2P2, HERC2P7, GOLGA8EP, GOLGA8S, GOLGA6L2, MIR4508, MKRN3, MAGEL2, NDN, PWRN4, PWRN2, PWRN3, PWRN1, NPAP1, SNRPN, SNHG14, SNURF, SNORD107, PWARSN, PWAR5, SNORD64, SNORD108, PWAR6, SNORD109B, SNORD109A, SNORD116-1, SNORD116-2, SNORD116-3, SNORD116-9, SNORD116-4, SNORD116-5, SNORD116-7, SNORD116-6, SNORD116-8, SNORD116-10, SNORD116-11, SNORD116-12, SNORD116-13, SNORD116-14, SNORD116-15, SNORD116-16, SNORD116-19, SNORD116-17, SNORD116-18, SNORD116-20, SNORD116-21, SNORD116-22, SNORD116-23, SNORD116-24, SNORD116-25, SNORD116-26, SNORD116-27, SNORD116-28, SNORD116-29, SNORD116-30, IPW, PWAR1, SNORD115-1, SNORD115-2, SNORD115-3, SNORD115-4, SNORD115-5, SNORD115-9, SNORD115-10, SNORD115-12, SNORD115-6, SNORD115-7, SNORD115-8, SNORD115-11, SNORD115-29, SNORD115-36, SNORD115-43, SNORD115-13, SNORD115-14, SNORD115-16, SNORD115-17, SNORD115-18, SNORD115-19, SNORD115-20, SNORD115-15, SNORD115-21, SNORD115-22, PWAR4, SNORD115-23, SNORD115-24, SNORD115-25, SNORD115-26, SNORD115-27, SNORD115-28, SNORD115-30, SNORD115-31, SNORD115-32, SNORD115-33, SNORD115-34, SNORD115-35, SNORD115-37, SNORD115-38, SNORD115-39, SNORD115-40, SNORD115-41, SNORD115-42, SNORD115-44, SNORD115-45, SNORD115-46, SNORD115-47, SNORD115-48, UBE3A, ATP10A, MIR4715, LINC02346, LINC00929, GABRB3, GABRA5, GABRG3, GABRG3-AS1, OCA2, HERC2, GOLGA8F, GOLGA8G, HERC2P11, HERC2P9, GOLGA8M, WHAMMP2, LOC100289656, PDCD6IPP2, APBA2, FAM189A1, NSMCE3, LOC100130111, TJP1, GOLGA8J, ULK4P3, GOLGA8T, LINC02249, CHRFAM7A, DNM1P50, GOLGA8R, LOC100288203, GOLGA8Q, ULK4P1, ULK4P2, GOLGA8H, ARHGAP11B, LOC100288637, HERC2P10, FAN1, MTMR10, TRPM1, MIR211, LINC02352, LOC283710, KLF13, OTUD7A, CHRNA7* |
| 80 | chr16 | p13.13 | 10,787,986 | 10,825,301 | 7 | -0.59091 | 5.29E-10 | *TEKT5* |
| 81 | chr17 | q25.3 | 79,686,397 | 79,945,570 | 16 | 0.425878 | 1.22E-13 | *SLC25A10, GCGR, MCRIP1, PPP1R27, P4HB, ARHGDIA, ALYREF, ANAPC11, PCYT2, NPB, SIRT7, MAFG, MAFG-AS1, PYCR1, MYADML2, NOTUM, ASPSCR1* |
| 82 | chr17 | q21.31 | 44,144,904 | 44,796,816 | 35 | -0.3532 | 5.69E-13 | *KANSL1, KANSL1-AS1, ARL17B, LRRC37A, ARL17A, NSFP1, LRRC37A2, NSF* |
| 83 | chr17 | p13.3 | 832,192 | 840,044 | 3 | -0.76757 | 9.16E-10 | *NXN* |
| 84 | chr18 | q23 | 74,221,768 | 74,289,678 | 13 | -0.59265 | 6.65E-19 | *LINC00908* |
| 85 | chr18 | q21.1 | 46,061,372 | 46,079,976 | 5 | -0.64149 | 9.47E-11 | *CTIF* |
| 86 | chr19 | q13.41 | 53,318,222 | 53,365,432 | 9 | 0.471631 | 6.01E-10 | *ZNF28, ZNF468, ZNF320* |
| 87 | chr19 | p13.3 | 5,832,884 | 5,840,036 | 3 | -0.8454 | 2.77E-10 | *FUT6* |
| 88 | chr19 | p13.2 | 12,538,730 | 12,545,513 | 3 | 0.790988 | 4.84E-10 | *ZNF443* |
| 89 | chr19 | p13.2 | 7,957,531 | 7,977,072 | 5 | -0.64286 | 3.71E-11 | *LRRC8E, MAP2K7* |
| 90 | chr19 | q13.42 | 54,188,930 | 54,193,007 | 3 | -0.70051 | 3.63E-10 | *MIR519C, MIR1283-1* |
| 91 | chr19 | q13.42 | 54,625,063 | 54,650,201 | 6 | 0.706203 | 8.71E-11 | *PRPF31, CNOT3* |
| 92 | chr19 | q13.41 | 52,127,719 | 52,148,589 | 4 | -0.59823 | 6.31E-10 | *SIGLEC5, SIGLEC14* |
| 93 | chr20 | q13.12 | 44,307,046 | 44,315,147 | 3 | -1.05459 | 6.37E-16 | *WFDC10B* |
| 94 | chr20 | p12.1 | 13,060,017 | 13,069,862 | 3 | -0.78223 | 2.87E-10 | *SPTLC3* |
| 95 | chr20 | p12.1 | 14,774,913 | 15,190,652 | 80 | -0.78202 | 8.94E-202 | *MACROD2, MACROD2-AS1* |
| 96 | chr20 | q13.12 | 45,764,881 | 45,809,227 | 10 | -0.57259 | 2.11E-14 | *EYA2, MIR3616* |
| 97 | chr21 | q22.13 | 38,297,322 | 38,308,143 | 3 | -0.81492 | 8.93E-10 | *HLCS* |
| 98 | chr21 | q22.3 | 44,949,257 | 45,071,430 | 25 | -0.31169 | 3.03E-13 | *HSF2BP, H2BFS, MIR6070* |
| 99 | chr21 | q22.3 | 44,953,212 | 45,085,165 | 27 | -0.25406 | 2.71E-10 | *HSF2BP, H2BFS, MIR6070, RRP1B* |
| 100 | chr22 | q11.21 | 18,889,039 | 18,984,519 | 14 | 0.672096 | 3.44E-32 | *DGCR6, PRODH, DGCR5* |
| 101 | chr22 | q13.31 | 45,579,409 | 45,583,183 | 3 | -0.78205 | 3.00E-10 | *NUP50, LOC105373064* |
| 102 | chr22 | q13.33 | 50,346,151 | 50,361,832 | 4 | 0.767625 | 7.90E-13 | *PIM3, MIR6821* |
| 103 | chr22 | q12.2 | 30,138,103 | 30,151,739 | 4 | 0.655037 | 1.48E-10 | *ZMAT5* |
| 104 | chr22 | q13.32 - q13.33 | 48,746,241 | 51,178,264 | 488 | -0.85405 | 4.900e-324 | *FAM19A5, LOC284933, MIR4535, LINC01310, C22orf34, MIR3667, BRD1, ZBED4, ALG12, CRELD2, PIM3, MIR6821, IL17REL, TTLL8, MLC1, MOV10L1, PANX2, TRABD, SELENOO, TUBGCP6, HDAC10, MAPK12, MAPK11, PLXNB2, DENND6B, PPP6R2, SBF1, ADM2, MIOX, LMF2, NCAPH2, SCO2, TYMP, ODF3B, KLHDC7B, SYCE3, CPT1B, CHKB-CPT1B, CHKB, CHKB-AS1, MAPK8IP2, ARSA, SHANK3, LOC105373100, ACR* |
| 105 | chr22 | q13.2 | 42,945,014 | 42,961,804 | 4 | -0.84768 | 7.45E-13 | *SERHL2* |
| 106 | chr22 | q13.33 | 51,137,326 | 51,170,223 | 10 | -1.04534 | 7.35E-47 | *SHANK3* |
| 107 | chr22 | q12.1 | 28,593,914 | 28,604,587 | 4 | 0.63623 | 1.00E-09 | *TTC28* |
| 108 | chr22 | q13.33 | 49,564,639 | 51,178,264 | 329 | -0.90018 | 4.900e-324 | *C22orf34, MIR3667, BRD1, ZBED4, ALG12, CRELD2, PIM3, MIR6821, IL17REL, TTLL8, MLC1, MOV10L1, PANX2, TRABD, SELENOO, TUBGCP6, HDAC10, MAPK12, MAPK11, PLXNB2, DENND6B, PPP6R2, SBF1, ADM2, MIOX, LMF2, NCAPH2, SCO2, TYMP, ODF3B, KLHDC7B, SYCE3, CPT1B, CHKB-CPT1B, CHKB, CHKB-AS1, MAPK8IP2, ARSA, SHANK3, LOC105373100, ACR* |
| 109 | chr22 | q11.21 | 18,991,215 | 19,007,211 | 4 | -0.79023 | 3.47E-12 | *DGCR5, DGCR9* |
| 110 | chrX | p22.33 | 298,354 | 339,035 | 20 | 0.510428 | 3.99E-17 | *PPP2R3B* |
| 111 | chrX | p22.33 | 70,397 | 505,732 | 113 | 0.312544 | 1.14E-15 | *PLCXD1, GTPBP6, LINC00685, PPP2R3B* |
| 112 | chrX | p22.33 | 1,446,802 | 1,559,802 | 39 | 0.386651 | 1.52E-11 | *IL3RA, SLC25A6, LINC00106, ASMTL-AS1, ASMTL* |
| 113 | chrX | p22.33 | 2,203,782 | 2,209,320 | 3 | -0.68061 | 6.46E-10 | *DHRSX* |
| 114 | chrX | p11.23 | 48,680,785 | 48,692,742 | 3 | -1.04028 | 1.40E-10 | *HDAC6, ERAS, PCSK1N* |
| 115 | chrX | p22.33 | 230,802 | 628,492 | 122 | 0.884013 | 4.900e-324 | *GTPBP6, LINC00685, PPP2R3B, SHOX* |
| 116 | chrX | q28 | 153,387,892 | 153,505,604 | 7 | -0.50813 | 4.64E-10 | *OPN1LW, OPN1MW2, OPN1MW, OPN1MW3, TEX28* |
| 117 | chrY | p11.32 | 248,354 | 289,035 | 20 | 0.510428 | 2.40E-16 | *PPP2R3B* |
| 118 | chrY | p11.2 | 9,641,061 | 9,650,194 | 3 | 1.282644 | 1.43E-15 | *TTTY22* |

## S5 Table: Primers sequences

**Designing primers on one significant gene for confirmation of each candidate CNV. The internal control gene is VPS29.**

| Cytoband | Gene | Forward Primer | Reverse Primer |
| --- | --- | --- | --- |
| 3p26.3 | *CNTN6* | GTGGGTGTGTATAATAATGAAGG | CAAGGGAACACAAACTATGC |
| 4q34.1-q35.2 | *LRP2BP* | TGGTTGATGGAATTCCTTACCTT | AGCTGCAATGCCTTATCCAC |
| 11q13.2-q13.4 | *SHANK2* | GGGCTCAGACATGAACG | CTGGACAGCACGAACC |
| 15q11.1-q13.3 | *GABRB3* | CCTGAAGGGACTATAAGTGG | GTTACCGGAGTGGAAAGG |
| 22q13.32-q13.33 | *SHANK3* | AACTTCCATGACCCTGAC | TGGGTTCACAGACAGATAAG |
| 12q24.11 | *VPS29*  *(Reference gene)* | GTCCCGAGATAAGAATGTCC | CAGAAAGTTGTGACTGTTGG |


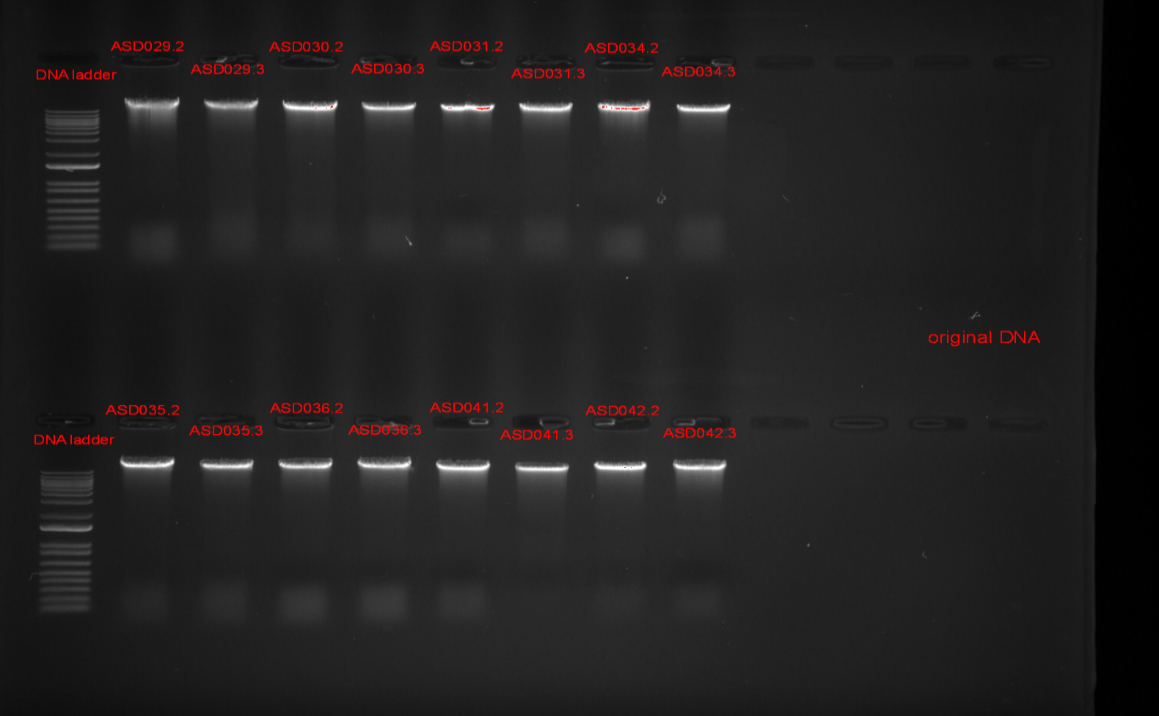


**B.**
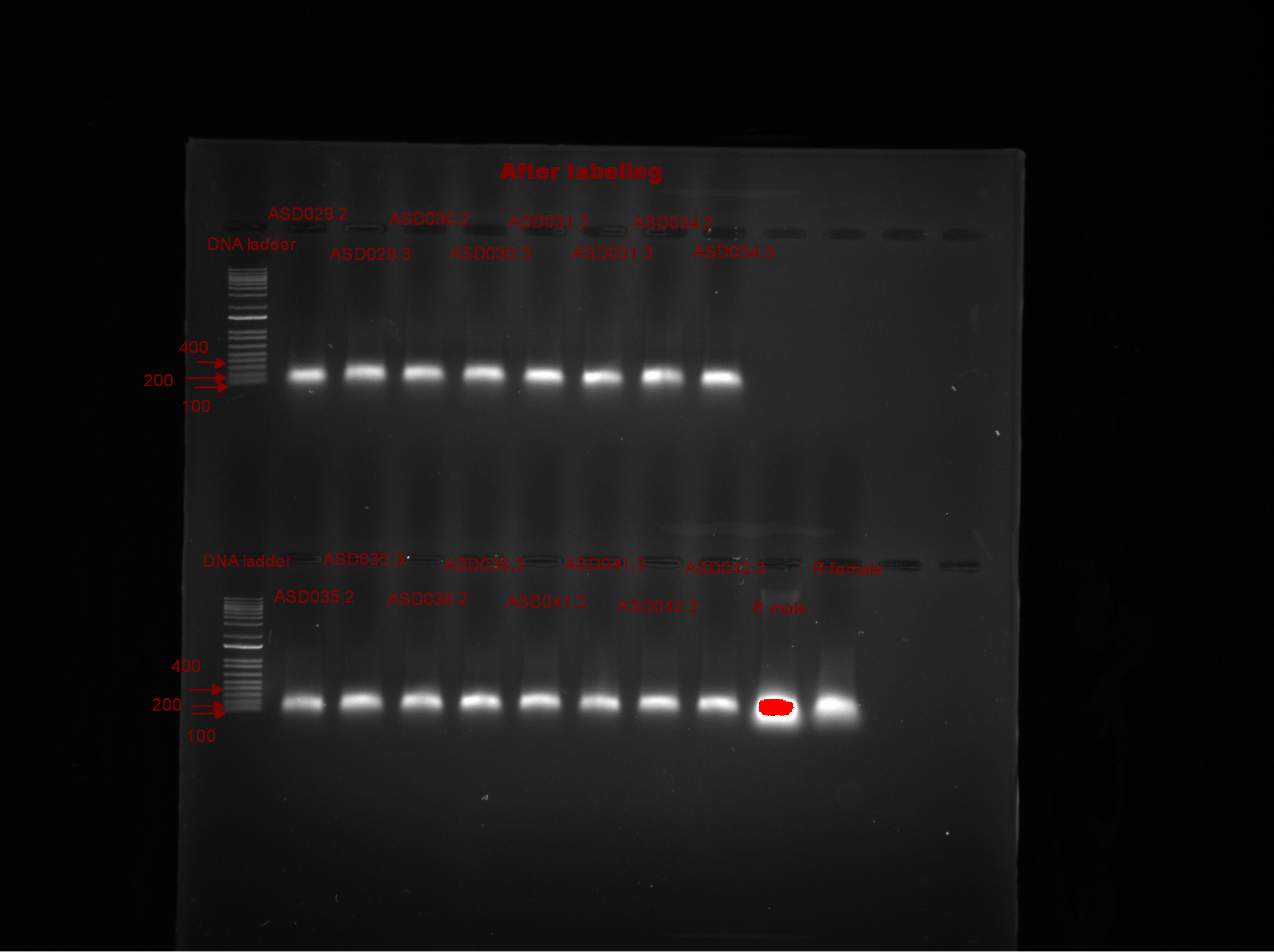


**S1 Figure:** Representative DNA quality check gel image to check the efficiency of restriction steps in aCGH experiments A. Original DNA B. Labeling products


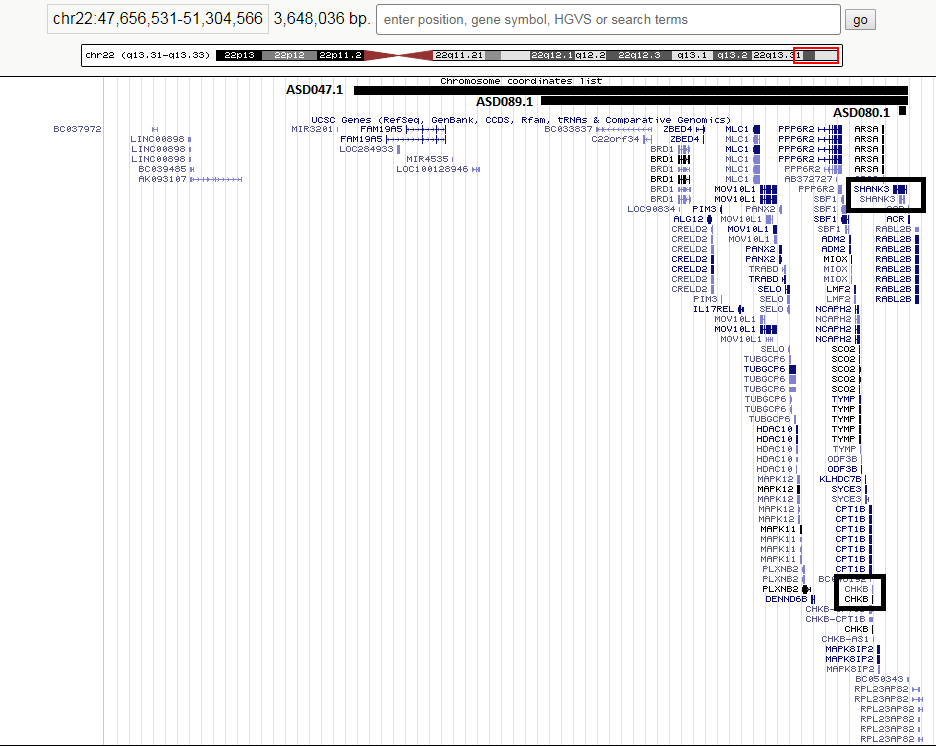


## S2 Figure: Genome browser view of the 3 specific CNVs

## Three patients (ASD047.1, ASD089.1 and ASD080.1) have a deletion CNV located in 22q13 with a part of *SHANK3* gene overlapping. The supplemental figure show CNVs have been found in 3 above patients with the containing gene list. These CNVs show the overlapping of a part of *SHANK3* gene
